# Development and *in-vivo* efficacy of Leicester-UTI1 phage cocktail that targets ESBL-associated *Klebsiella* from urinary tract infections

**DOI:** 10.1128/spectrum.03952-25

**Published:** 2026-06-24

**Authors:** Rizka O. A. Jariah, Jennifer Schofield, Kevin West, Karen D. Adler, Slawomir Michniewski, Caitlin Wildsmith, Eleanor Jameson, Lucy Onions, Faizal Patel, Anusha Saravanan, Hasan Yesilkaya, Spyridon Megremis, Andrew D. Millard, Martha R. J. Clokie, Melissa E. K. Haines

**Affiliations:** 1 Becky Meyer Center for Phage Research, Division of Microbial and Infection, School of Biological and Biomedical Sciences, College of Life Sciences, University of Leicester 98072, Leicester, United Kingdom; 2 Medical Laboratory Technology, Faculty of Vocational, Universitas Airlangga 148005, Surabaya, Indonesia; 3 Leicester Drug Discovery and Diagnostics, University of Leicester 4488, Leicester, United Kingdom; 4 University Hospitals of Leicester NHS Trust 4490, Leicester, United Kingdom; 5 School of Environmental and Natural Sciences, Bangor University 151724, Bangor, United Kingdom; 6 Division of Biomedical Services, University of Leicester 4488, Leicester, United Kingdom; 7 Division of Microbiology and Infection, School of Biological and Biomedical Sciences, College of Life Sciences, University of Leicester 98072, Leicester, United Kingdom; University of California San Diego, La Jolla, California, USA; University of Ottawa, Ottawa, Canada

**Keywords:** immune response, antimicrobial resistance, phage therapy, urinary tract infection

## Abstract

Uropathogenic *Klebsiella pneumoniae* strains are of major concern in the context of antimicrobial resistance, which has sparked interest in phage therapy as an alternative or complement to antibiotics. To address this, we developed and evaluated the Leicester-UTI1 phage cocktail, composed of six obligate lytic phages. Leicester-UTI1 phage cocktail demonstrated an *in vitro* efficacy of 80% against a panel of 30 diverse extended-spectrum β-lactamase (ESBL) and non-ESBL *Klebsiella* clinical isolates, as well as the reference *K. pneumoniae* Top52 strain. To assess *in vivo* activity, catheterized female C57BL/6J mice were infected transurethrally with *K. pneumoniae* Top52 and treated with a single dose of the purified Leicester-UTI1. Over a 7-day observation period modeling an acute urinary tract infection (UTI), the phage treatment significantly reduced or eradicated the *K. pneumoniae* infection in the urine, bladder, and kidneys, with effects observable as early as 4 h post-treatment. Phage recovery data indicated active replication in the urine, with no evidence of systemic dissemination, as no phage were detected in the spleen or blood. No emergence of phage resistance was observed. Phage treatment also reduced bladder inflammation, decreased pro-inflammatory IL-6, and increased anti-inflammatory IL-10 protein levels, indicating a positive immunomodulatory effect and enhanced infection resolution. Overall, the Leicester-UTI1 phage cocktail is a promising therapeutic agent for the treatment of *Klebsiella pneumoniae* UTI.

**IMPORTANCE:**

The emergence of antibiotic resistance, coupled with a slow development of new antimicrobials, has renewed the interest in phage therapy, including for the treatment of urinary tract infections (UTIs). Despite the growing number of animal studies evaluating phage therapy, UTIs remained relatively understudied in this context. Here, we established a robust acute UTI murine model and used it to assess the efficacy of our optimized Leicester-UTI1 phage cocktail targeting extended-spectrum β-lactamase-associated *Klebsiella*. We found that a single transurethral administration of Leicester-UTI1 was sufficient to consistently eradicate bacterial infection in the urine, bladder, and kidneys. Further investigation also demonstrated that the phage exhibited immunomodulatory effects that supported infection resolution. Overall, our study provides strong evidence for the therapeutic potential of phages against UTI pathogens and highlights their promise as an alternative approach to antibiotics.

## INTRODUCTION

Urinary tract infections (UTIs) are among the most prevalent bacterial infections worldwide, occurring in both the community and healthcare settings (1, 2). They can affect men and women of all ages, but due to anatomical differences in the genitourinary tract, infection is more common in women (3). In 2019, over 404.6 million individuals experienced UTIs globally, contributing to approximately 230,000 deaths (4) alongside substantial economic and healthcare costs (2). Uncomplicated UTIs occur in otherwise healthy individuals with no structural or functional abnormalities of the urinary tract, whereas complicated UTIs are associated with factors that increase the risk of treatment failure, such as urinary obstruction, catheterization, or immunosuppression. UTIs are categorized by infection site as lower UTIs (cystitis), affecting the bladder, or upper UTIs (pyelonephritis), involving the kidneys (5, 6). Several risk factors have been associated with UTIs, including being female, sexual activity, vaginal infection, prior UTIs, genetic susceptibility, and diabetes (3, 7). Nosocomial UTIs are also common (~30%) either due to catheter-associated infections or transmission outbreaks that occur between patients in hospitals (3, 8).

There is an increased prevalence of UTI cases caused by *Klebsiella pneumoniae* producing extended-spectrum β-lactamases (ESBL); the most frequent acquired resistance mechanism in *Klebsiella* (8, 9). Medical treatment of ESBL-producing *K. pneumoniae* is therefore becoming increasingly difficult. Lack of rapid and reliable diagnostic tests to identify beta-lactamase resistance has led to the missed or late drug prescription, which eventually contributes to the widespread resistance phenotype (10, 11). The development of novel treatment strategies is clearly needed, and one potent and promising alternative is to use bacteriophages or phages, which are natural bacterial predators (12).

The uropathogenic *Klebsiella pneumoniae*, *Escherichia coli*, and *Pseudomonas aeruginosa* species are major contributors to the growing global antimicrobial resistance problem (13–15), significantly impacting clinical management by delaying effective treatment and increasing morbidity (4, 16). The emergence of multidrug resistance in these strains is primarily driven by the overuse and frequent administration of antibiotics, often in the absence of proper diagnostic confirmation (17).

To address UTIs, *in vivo* research, *in vitro* research, case reports, and clinical trials have been conducted to assess the use of phage therapy (18–20). *In vivo* models are crucial for evaluating the effects of phage therapy before its application in clinical trials. Factors such as the urinary environment, host immune response, and uropathogenic characteristics influence phage efficacy and pharmacodynamics; therefore, they should be considered carefully (21, 22). Nonetheless, it is understudied in the context of UTIs (23).

Recent work by Singh et al. (24) has provided useful insight into the *in vivo* efficacy of phage therapy against *Klebsiella pneumoniae* UTIs, particularly in relation to dosing strategies and routes of administration, with outcomes primarily assessed through bacterial detection in urine.

Our study builds on this by extending the analysis to a more comprehensive assessment of infection dynamics within the urinary tract. Specifically, we developed an optimized six-phage cocktail with expanded host range across a diverse panel of clinical isolates and evaluated efficacy across multiple infection sites, including urine, bladder, and kidneys. In addition, we incorporated histopathological and cytokine analyses to assess tissue-level infection and host response. This approach provides further insight into how phage therapy influences not only bacterial burden but also inflammation and infection resolution within the urinary tract.

We aimed to address the need for developing effective and safe phage therapy against *Klebsiella pneumoniae* in acute, uncomplicated UTIs. We present the development, composition, *in vitro* efficacy, and *in vivo* properties of the Leicester-UTI1 phage cocktail in our UTI model. Last, we also evaluated the phage therapy effect at the intracellular level, through microbiology assessment and histopathology.

## RESULTS

### Expanding phage cocktail breadth against diverse UTI-associated *Klebsiella* isolates

A panel of 30 clinically relevant UTI-associated *Klebsiella* isolates, including both ESBL and non-ESBL strains from diverse UK sources, was used in this study (Table 2). These comprised five species, predominantly *K. pneumoniae* (26/30), with additional representation from *Klebsiella michiganensis*, *Klebsiella oxytoca*, *Klebsiella variicola*, and *Klebsiella quasipneumoniae* (Fig. S1a; Table S1). Phylogenetic analysis confirmed species-level clustering with strong internal node support (>70%; Fig. S1a). The collection was highly diverse and encompassed 22 sequence types, including ST147 isolates associated with hypervirulence and resistance plasmids (Table S1) (25). Capsule typing further highlighted surface variability, with 25 distinct K-loci and 7 O-loci identified. Genomic antimicrobial resistance (AMR) profiling revealed 89 resistance determinants across the panel (Fig. S1b; Table S2), with 40% (12/30) carrying ESBL genes, 36.7% (11/30) carbapenemase producers, and five strains harboring both. This level of heterogeneity reflects the clinical challenge posed by *Klebsiella* UTIs and underscores the need for phage cocktails with a broad and robust host range.

**Fig 1:**
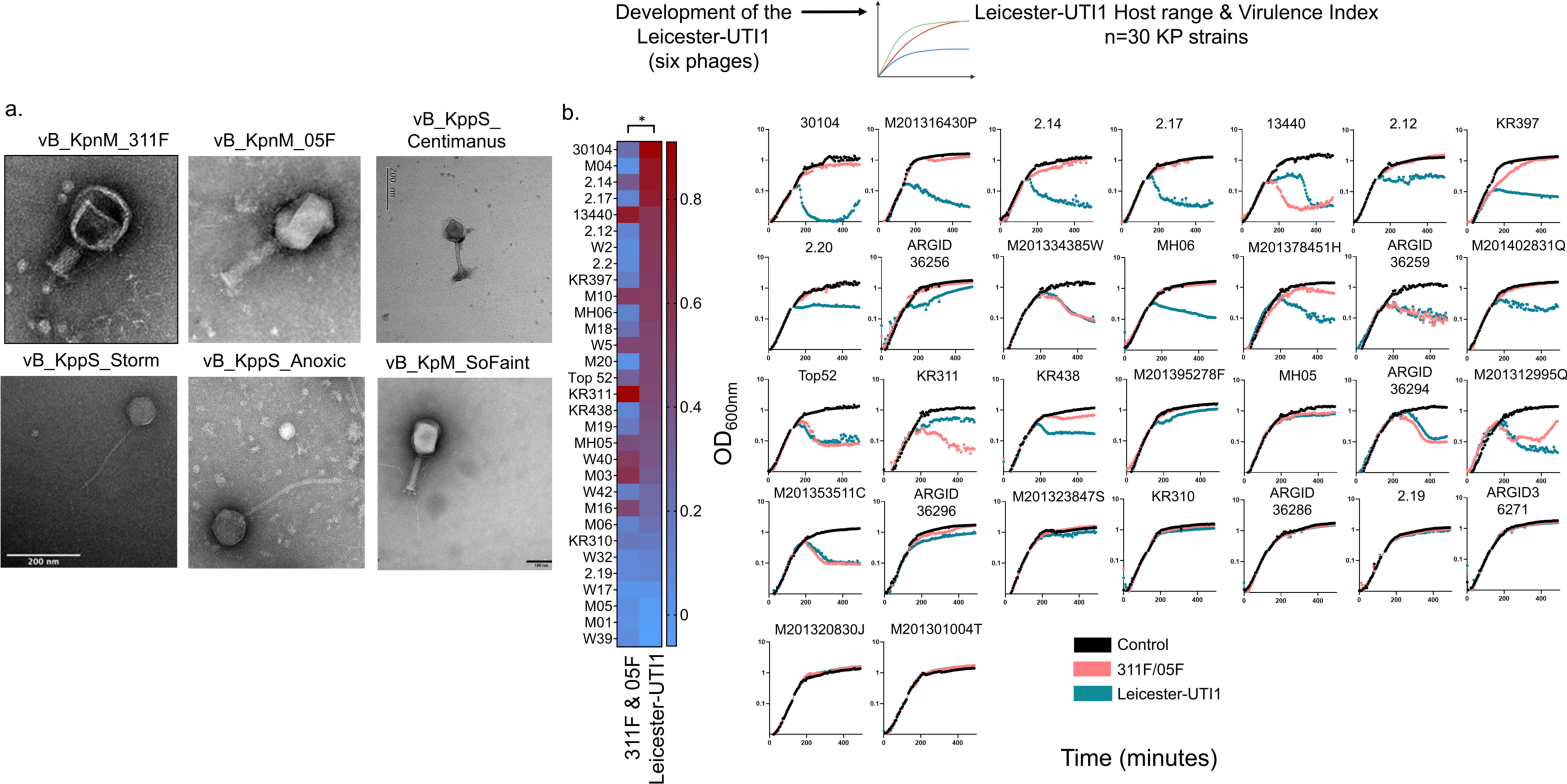
Leicester-UTI1 phage cocktail efficacy against ESBL-producing *Klebsiella* species. (**a**) Morphological properties of phages: 311F, 05F, Centimanus, Storm, Anoxic, and SoFaint. (**b**) Killing efficacy comparison of phages 311F and 05F and Leicester-UTI1. Heat-map of local virulence index (vi) and killing curves data shows the result of 311F and 05F and Leicester-UTI1 (05F, 311F, Centimanus, Storm, Anoxic, SoFaint) against 29 ESBL-producing *Klebsiella* clinical isolates and *K. pneumoniae* Top52. The latter has a better efficacy against strains tested. The local vi score was categorized into low (local vi ＜0.2), medium (0.2 ≤ local vi ≥ 0.5), and high (local vi > 0.5). An effective killing is described as having medium and high local vi score. Cocktail 311F and 05F and Leicester-UTI1 to have 40% and 80% across the clinical isolate collection, respectively. *Significance difference between 311F and 05F and Leicester-UTI1 (*P < 0.05).

To address this, the Leicester-UTI1 cocktail (Table 1) was developed through a stepwise optimization process. We have previously described phage isolation, characterization (Fig. 1a), and individual host range using local *virulence index* (*vi*) and direct spot testing (Table S3) (26, 27). Briefly, effective killing was defined as medium to high *vi* (≥0.2). An initial two-phage combination (311F and 05F) showed 53% activity against ESBL-producing isolates in earlier work (26) but achieved 40% (12/30) coverage against the expanded panel used here (Fig. 1b; Table S3).

**TABLE 1:** Phages and original propagation hosts used in this study

Phages	Source of isolation	Accession no.	Host strain	Host source	Accession no.
vB_KpnM_311F	Sewage	LR877331	*K. pneumoniae* KR311	Leicester Royal Infirmary, UK	ERS29686510
vB_KpnM_05F	Sewage	LR746310	*K. pneumoniae* MH05	Leicester Royal Infirmary, UK	ERS29686511
vB_KppS_Anoxic	Sewage-anoxic sludge	LR881109	*K. pneumoniae* 30104	DSMZ Culture Collection (Human Blood)	ERS29671980
vB_KpM_SoFaint	Sewage-mixed liquor	LR881141	*K. pneumoniae* 13440	NCTC Culture Collection (Clinical)	UGKX00000000
vB_KpM_ Centimanus	Sewage	OZ240615	*K. pneumoniae* 13440	NCTC Culture Collection (Clinical)	UGKX00000000
vB_KppS_Storm	Sewage—storm tank	LR881112	*K. pneumoniae* 30104	DSMZ Culture Collection (Human Blood)	ERS29671980

**Fig 2:**
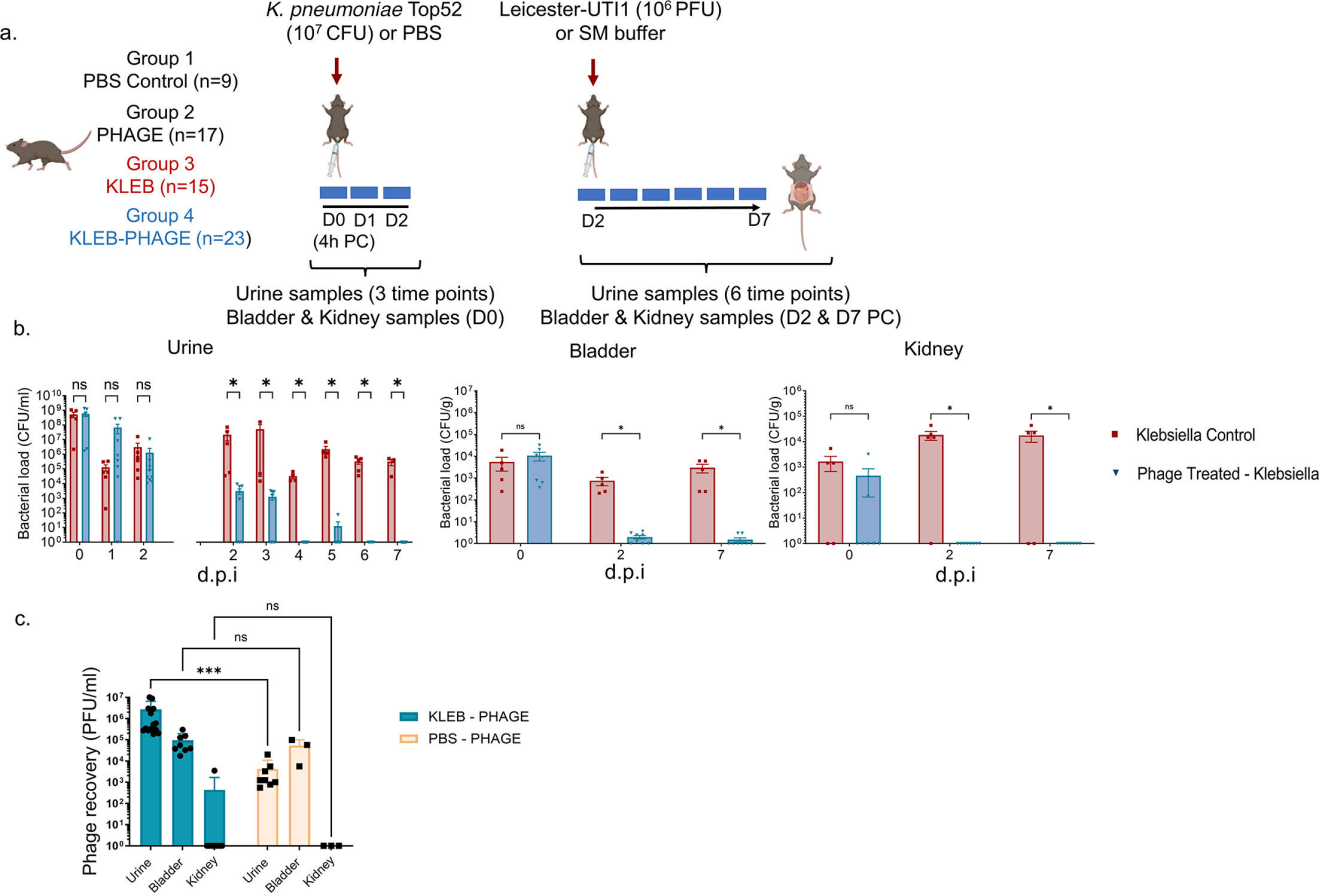
Phage therapy evaluation in UTI murine model. (**a**) *In vivo* experimental design to evaluate Leicester-UTI phage cocktail to treat Klebsiella infection with bacterial inoculation at day 0 and treatment at day 2 (48 h post-infection). (**b**) Bacterial burden in urine from day 0 to day 7 shows significant and consistent bacterial reduction in the phage-treated group compared to the control; bacterial burden in bladder lysate samples days 0, 2, and 7 shows consistent clearance in the phage-treated group; bacterial burden in kidney homogenates days 0, 2, and 7 shows total clearance of Top52 in the phage-treated group. (**c**) Phage recovery 4 h post-phage cocktail treatment on day 2 (*P < 0.05; ***P < 0.001; ns, not significant).

To improve coverage, additional phages were selected specifically to target strains resistant to the initial combination. Incorporation of four phages (Centimanus, Storm, Anoxic, and SoFaint) resulted in the six-phage Leicester-UTI1 cocktail, increasing host range to 80% (24/30 isolates; Fig. 1b; Table S3). Leicester-UTI1 comprises six obligately lytic phages lacking integrase genes (Table 1; Data S1) (26, 27). The cocktail spans two families (Demerecviridae and Straboviridiae) and four genera (Shenzhenvirus, Jiadovirus, Slopekvirus, and Sugarlandvirus; Fig. S2). Morphologically, TEM analysis classified phages 311F, 05F, SoFaint, and Centimanus as myoviruses, and Anoxic and Storm as siphoviruses (Fig. S2; Data S1). The final cocktail was endotoxin-reduced (Data S2) prior to use in *in vivo* experiments (Fig. 2a).

### Leicester-UTI1 phage cocktail disrupts *K. pneumoniae* Top52 colonization

Endotoxin-purified Leicester-UTI1 (Fig. S3) was tested for its safety and efficacy *in vivo* by assessing the microbiology, immunopathology, and cytokine expression (Fig. S4a). Additionally, we also checked for potential phage resistance. To do this, we established an acute UTI model in C57BL/6J female mice by transurethral inoculation with *K. pneumoniae* Top52 (28) (Fig. S4b). The established model exhibited consistent bacterial shedding in urine and bladder tissue and ascended to the kidney by day 7 (Fig. S4b). A bacterial inoculum of 1 × 10^7^ CFU in 50 μL, as previously described (28), resulted in a consistent infection, yielding bacterial loads higher than 10^4^ CFU/g in both homogenized bladder and in the urine up to day 7 post-infection.

This model was subsequently used to evaluate the efficacy of the Leicester-UTI1 phage cocktail. Mice were divided into four experimental groups: PBS Control (PBS and SM-Buffer control) group, PHAGE (phage cocktail only), KLEB (*Klebsiella* infection control) group, and KLEB-PHAGE (*Klebsiella* and phage cocktail treatment) group (Fig. 2a). Four hours after bacterial or PBS administration, 5 (KLEB); 7 (KLEB-PHAGE); 5 (PHAGE); 3 (CONTROL) mice were sacrificed to confirm successful bacterial colonization by determining bacterial load in the organs (bladder and kidneys). The Leicester-UTI1 phage cocktail was given 48 h (day 2) post-infection at 10^6^ PFU in 50 μL, corresponding to a multiplicity of infection (MOI) of 0.1 based on the initial bacterial inoculum, to both the KLEB-PHAGE and PHAGE groups. As a control, SM-buffer was administered to the CONTROL and KLEB groups. After 4 h, 5 (KLEB); 8 (KLEB-PHAGE), 6 (PHAGE); 3 (CONTROL) mice were sacrificed for analysis, while the remaining mice were monitored until day 7. At each time point, the primary outcome measured was the average bacterial burden in the bladder and kidneys. In addition, urinary bacterial shedding was monitored daily from day 0 to day 7 as a non-terminal indicator of infection (Fig. 2b).

*Klebsiella* levels in urine were monitored in all four groups (Fig. 2b). As expected, no bacteria were detected in the PHAGE and CONTROL groups (data not shown). In contrast, in the infected mice without phage treatment (KLEB group), bacterial abundance ranged from 10^6^ to 10^9^ CFU/mL on day 0 and approximately 10^4^ to 10^6^ CFU/mL by day 2, remaining above 10^4^ CFU/mL throughout the course of infection. Administration of Leicester-UTI1 on day 2 resulted in a rapid reduction in *Klebsiella* levels within 4 h, with significantly lower bacterial counts in the urine from phage-treated mice on days 3 to 5, and no detectable bacteria on days 6 and 7 (Fig. 2b).

Bladder and kidney homogenates were also analyzed for *Klebsiella* abundance on day 0, 2, and 7 (Fig. 2b). In the absence of phage treatment, *Klebsiella* persisted at approximately 10^4^ CFU/g in the bladder samples until day 7 (Fig. 2b). On the contrary, in the KLEB-PHAGE group, the bacterial burden dropped significantly on day 2 by ~3 log and continued to decline to 10^1^ CFU/g by day 7 (Fig. 2b). In the kidneys, *Klebsiella* was detected as early as day 0 of infection (4 h post-infection) at 10^3^ CFU/g in both Top52 infected groups, but after day 2 *Klebsiella* levels dropped in the phage-treated group by ~3 log. In contrast, the levels were maintained at 10^3^–10^4^ CFU/g in the KLEB group throughout the time course. These results indicate that a single application of phage treatment was sufficient to eliminate the infection within the mouse urinary tract. No *Klebsiella* was observed in the blood samples or spleen lysates for any of the mice groups, showing that no systemic infection occurred during the time course.

Phage titers were assessed 4 h after transurethral administration (Fig. 2c). A significant difference in phage recovery titer was observed between the PHAGE group and the KLEB-PHAGE group for the urine samples, with 10^4^ PFU/mL and 10^6^ PFU/mL, respectively. This implies that phages propagate in the urine when bacteria are present. Although in the bladder, both groups exhibited a phage concentration of 10^5^–10^6^ PFU/mL at 4 h post-treatment, with no significant difference observed between the groups. Phages were also detected in one of the KLEB-PHAGE mice kidney samples at ~10^3^ PFU/mL but not in PHAGE group mice. As expected, no phage was detected in the KLEB or CONTROL groups.

### Recovered *K. pneumoniae* Top52 colonies are still susceptible to the phage cocktail

To evaluate if resistance to the phage cocktail emerged i*n vivo*, bacteria were isolated from the bladders of two mice on day 7. Three isolates were randomly selected and challenged against the Leicester-UTI1 phage cocktail to assess their susceptibility using a killing assay and local *vi* (Fig. S4c). All three isolates, 191, 197, and 200, exhibited comparable sensitivity to the phage cocktail as the parental *K. pneumoniae* Top52 strain, with local *vi* scores of 0.30, 0.32, 0.34, and 0.34, respectively. Furthermore, no significant differences were observed in growth rates and doubling times between the recovered isolates and the original Top52 strain (Table S4).

### The Leicester-UT1 phage cocktail reduces inflammation

**Fig 3:**
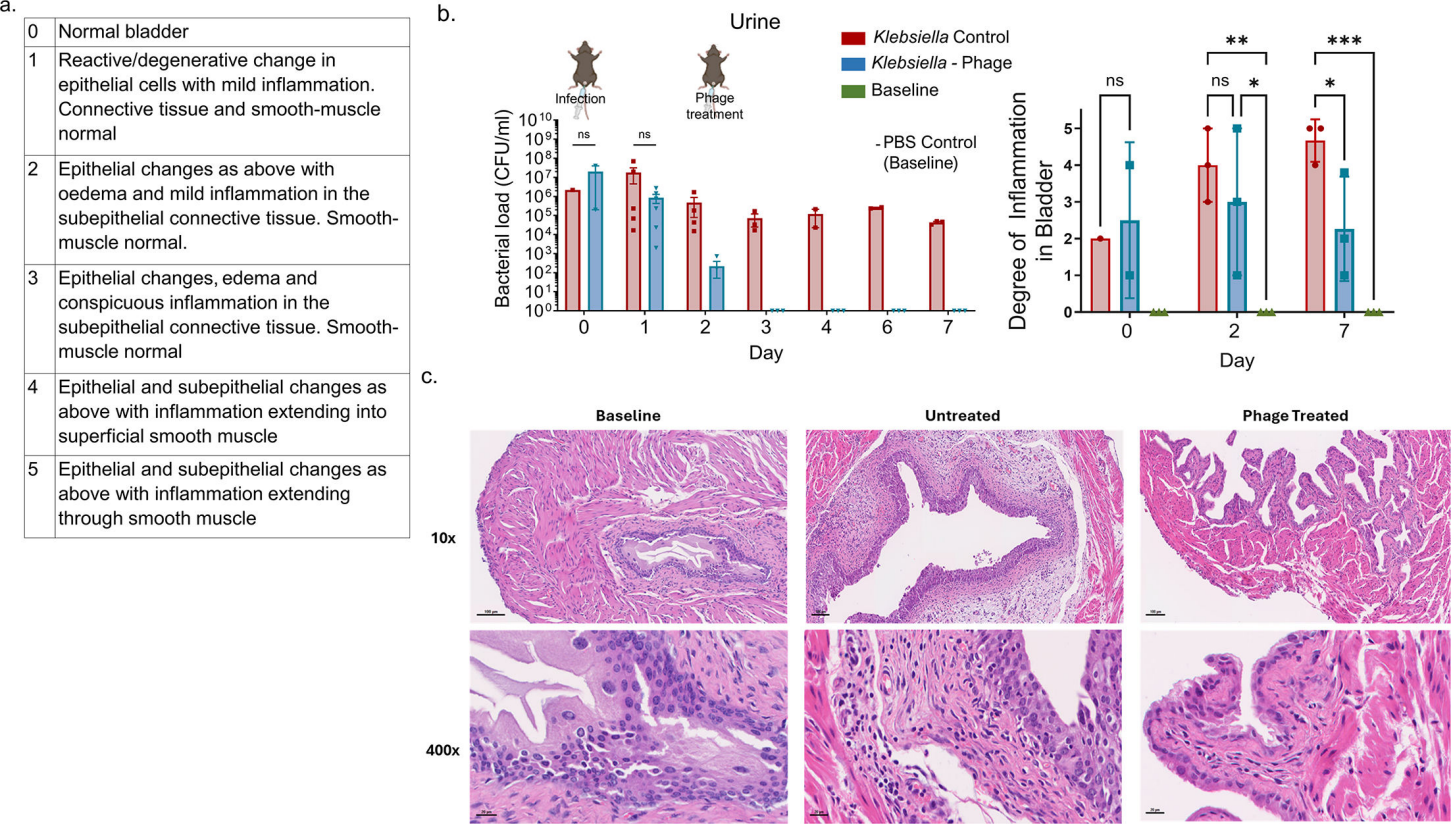
Phage treatment reduces infection-induced inflammation and modulates cytokine expression. (**a**) Histopathology grading scale for the degree of inflammation in the bladder tissue. Ranging from 0 to 5, with 0 representing no inflammation and 5 representing the most severe and extending inflammation. (**b**) Bacterial loads in the urine samples taken from histology mice group day 0 until day 7, and degree of inflammation mean score in the bladder on days 0, 2 (4 h post-phage treatment), and 7 (*P < 0.05, **P < 0.01, and ***P < 0.001; ns, not significant). (**c**) Representative of hematoxylin and eosin staining of bladder tissue at 10× and 400× magnification (top–down, respectively). From left to right, baseline inflammation score: 0; untreated mice bladder day 7 inflammation score: 5; phage-treated mice bladder day 7 (post 5 days of phage treatment) inflammation score: 1 (all histology pictures are in Fig. S5).

Histopathology analysis was performed to assess the degree of inflammation in bladder tissue during infection and following phage treatment (Fig. S5). The detailed description of the inflammation score is depicted in Fig. 3a. The PBS Control group served as a baseline reference, exhibiting an inflammation score of zero at all time points (Fig. 3b). At 4 h post-infection (day 0), inflammation scores ranged from 1 to 4 in both the KLEB and KLEB-PHAGE groups. After 4 h post-phage treatment (day 2), the KLEB group had increased the severity, exhibiting an inflammation score mean of 4, whereas the KLEB-PHAGE group exhibited a mean score of 3. By day 7, the difference became more pronounced with mean inflammation scores of 4.7 and 2.3 for the KLEB and KLEB-PHAGE groups, respectively. At the final time point (day 7), all bladder tissues from KLEB group had inflammation, which extended into smooth muscle, whereas in KLEB-PHAGE group, the smooth muscle remained normal and unaffected (Fig. 3b and c; Fig. S5). The observed reduction in bladder inflammation in KLEB-PHAGE group also correlated with the reduced bacterial load in urine samples, indicating that phage therapy contributed to mitigating infection-induced tissue damage (Fig. 3b).

### Pro-inflammatory (IL-6, IFN-gamma, and TNF-alpha) and anti-inflammatory (IL-10) cytokine secretion from serum and bladder lysate

**Fig 4:**
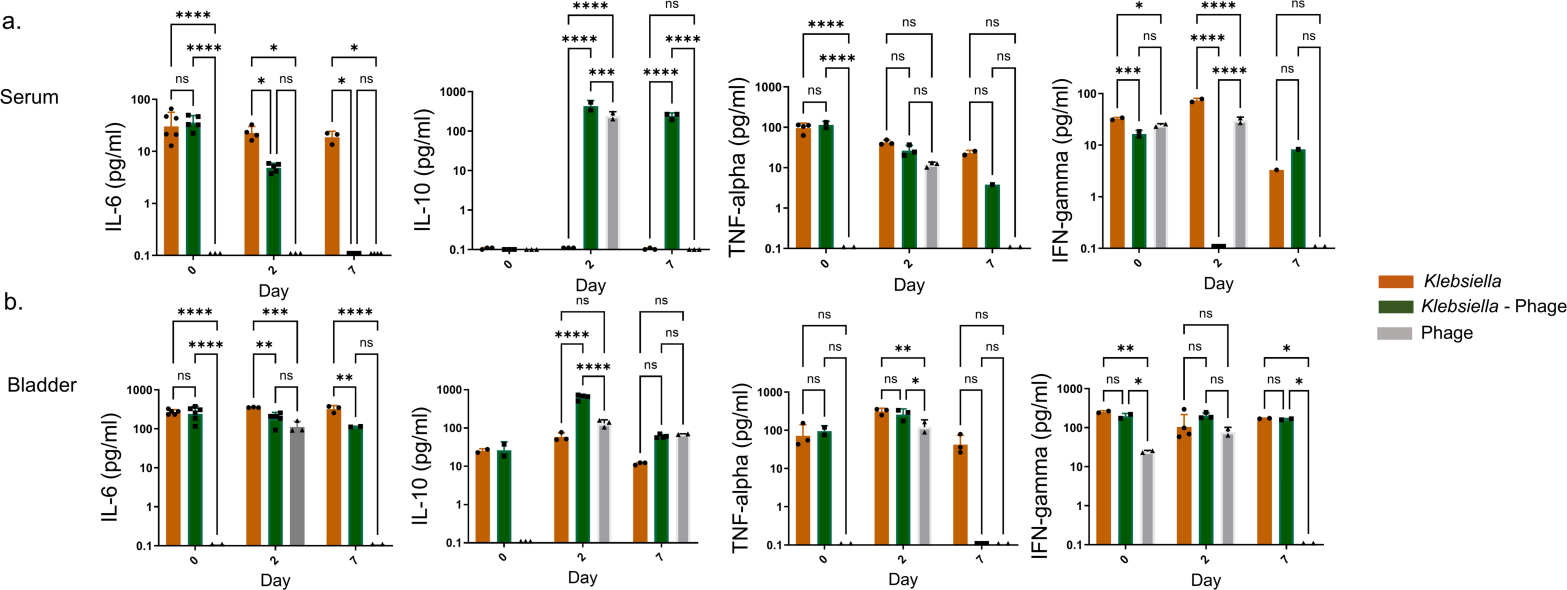
Cytokine expression to phage therapy. (**a**) Expression of IL-6, IL-10, TNF-alpha, and IFN-gamma (left-to-right) in pg/mL from serum samples (top) on day 0 (4 h post-infection), day 2 (48 h post-infection or PBS control and 4 h post-phage treatment or SM-Buffer), and day 7. (**b**) Expression of IL-6, IL-10, TNF-alpha, and IFN-gamma (left-to-right) from bladder lysate samples (bottom) on day 0 (4 h post-infection), day 2 (48 h post-infection or PBS control and 4 h post-phage treatment or SM-Buffer), and day 7 (*P < 0.05, **P < 0.01, ***P < 0.001, and ****P > 0.0001).

To determine the impact of the phage treatment on the inflammatory response, serum and bladder lysate samples were analyzed for the expression of pro-inflammatory cytokines (IL-6, IFN-gamma, and TNF-alpha) and the anti-inflammatory cytokine (IL-10; Fig. 4a and b). From the serum samples (Fig. 4a), it was observed that the level of all pro-inflammatory cytokines: IL-6, TNF-alpha, and IFN-gamma were all elevated 4 h post *K. pneumoniae* Top52 inoculation. By day 2, IL-6 and IFN-gamma concentrations in the KLEB group were significantly higher than in the KLEB-PHAGE group (Fig. 4a, *P* < 0.05). IL-6 levels remained elevated in the KLEB group through to day 7, whereas TNF-alpha and IFN-gamma showed variable patterns. The elevated IFN-gamma expression was also observed in the PHAGE group, which received PBS at day 0; this was likely associated with the catheterization procedure rather than bacterial. Interestingly, anti-inflammatory IL-10 expression in serum was undetectable on day 0 in all groups but became significantly elevated by day 2 in both groups receiving phage (KLEB-PHAGE group and PHAGE group). The level of IL-10 in the phage-treated group remained high until day 7.

A similar cytokine profile was observed in the bladder lysate (Fig. 4b), although overall cytokine concentrations were generally 10- to 100-fold higher than those detected in serum (Fig. 4a). IL-6 expression was significantly higher in the KLEB group compared to the KLEB-PHAGE group on day 2 (Fig. 4b), consistent with a reduction in local inflammation following phage treatment. In contrast, as observed in the serum, the levels of TNF-alpha and IFN-gamma did not differ significantly between these groups. IL-10 expression in bladder lysate followed a similar trend to the serum, with markedly higher levels in the KLEB-PHAGE group than in the KLEB group. IFN-gamma expression remained persistently elevated in both KLEB and KLEB-PHAGE groups (Fig. 4b).

## DISCUSSION

Although many *Klebsiella* phages have been isolated and characterized (26, 27), their clinical translation to combat *Klebsiella* UTIs remains remarkably underexplored, despite *Klebsiella* being identified as the second leading cause of UTIs after *E. coli* (21) (29). Surprisingly, very few studies have evaluated the efficacy of *K. pneumoniae* phage therapy *in vivo*, or in animal models (24, 30). To address this gap, our study aimed to provide a comprehensive evaluation of both the efficacy and safety of *Klebsiella*-targeting phage cocktail, Leicester-UTI1, in a robust *in vivo* model. Using a validated *K. pneumoniae* acute UTI mouse model, we assessed phage therapy outcomes by measuring bacterial clearance and phage propagation in the *in vivo* system. As such, in addition to this microbiology data, to determine how phages interact with a mammalian immune system, we examined infection-induced inflammation using histopathological analysis and cytokine expression profiling.

In this study, a previously developed phage cocktail from our laboratory that targets ESBL-producing clinical UTI *Klebsiella* isolates was further developed to ensure clinical relevance in terms of the spectrum of activity on relevant strains, particularly for individuals with limited antibiotic treatment options. The clinical isolates were obtained from multiple locations across the UK—Leicester, Manchester, Bristol, and Public Health Wales—to represent a broad diversity of clinically relevant and prevalent strains (Table 2). Earlier *in vitro* studies from our group identified an optimized phage cocktail, consisting of phage 311F and 05F, that targeted ESBL-producing *Klebsiella* strains, exhibiting a 53% (10/19) host range coverage (26). Building upon this, we developed an expanded cocktail, Leicester-UTI1, composed of six phages (311F, 05F, Centimanus, Storm, Anoxic, and SoFaint). The comparative efficacy of both cocktails was evaluated using a phage killing assay (PKA) and quantified as local *vi* score, which is a quantitative method that compares the area under the growth curve of bacteria following phage addition to a standard bacteria-only growth curve (31). Phage cocktail activity is generally assessed using two key criteria, the first being the breadth of activity, which refers to the number of isolates the cocktail can infect, to identify those capable of targeting a wide range of strains. The second is the depth or strength of activity, which measures the ability to reduce bacterial numbers. Although we value both parameters for clinical applications, it is essential to target as many clinical strains as possible, and thus, we have prioritized this parameter over a cocktail with a narrower spectrum (32).

**TABLE 2:** *Klebsiella* spp. clinical strains used in the host range study^*a*^

No	Lab strains name	Species	Source	Accession no.
1	M201301004T	*K. pneumoniae*	Manchester Royal Infirmary	ERS29686495
2	M201312995Q	*K. pneumoniae*	Manchester Royal Infirmary	ERS29686496
3	M201316430P	*K. michiganensis*	Manchester Royal Infirmary	ERS29686497
4	M201320830J	*K. pneumoniae*	Manchester Royal Infirmary	ERS29686498
5	M201323847S	*K. pneumoniae*	Manchester Royal Infirmary	ERS29686499
6	M201334385W	*K. pneumoniae*	Manchester Royal Infirmary	ERS29686500
7	M201353511C	*K. pneumoniae*	Manchester Royal Infirmary	ERS29686501
8	M201378451H	*K. oxytoca*	Manchester Royal Infirmary	ERS29686502
9	M201395278F	*K. pneumoniae*	Manchester Royal Infirmary	ERS29686503
10	M201402831Q	*K. pneumoniae*	Manchester Royal Infirmary	ERS29686504
11	ARGID 36256	*K. pneumoniae*	Public Health Wales	Genome not published
12	ARGID36271	*K. variicola*	Public Health Wales	Genome not published
13	ARGID 36,286	*K. pneumoniae*	Public Health Wales	Genome not published
14	ARGID 36,296	*K. pneumoniae*	Public Health Wales	Genome not published
15	ARGID 36,259	*K. pneumoniae*	Public Health Wales	Genome not published
16	ARGID 36,294	*K. pneumoniae*	Public Health Wales	Genome not published
17	KR 310	*K. pneumoniae*	Leicester Royal Infirmary	ERS29686505
18	KR 397	*K. pneumoniae*	Leicester Royal Infirmary	ERS29686506
19	KR 438	*K. pneumoniae*	Leicester Royal Infirmary	ERS29686507
20	MH 06	*K. pneumoniae*	Leicester Royal Infirmary	ERS29686508
21	P2.12	*K. pneumoniae*	North Bristol NHS Trust	ERS29408120
22	P2.14	*K. pneumoniae*	North Bristol NHS Trust	ERS29408122
23	P2.17	*K. pneumoniae*	North Bristol NHS Trust	ERS29408124
24	P2.19	*K. quasipneumoniae sub.similipneumoniae*	North Bristol NHS Trust	ERS29686509
25	P2.20	*K. pneumoniae*	North Bristol NHS Trust	ERS29408126
26	13,440	*K. pneumoniae*	National Collection of Type Cultures (NCTC)	UGKX00000000
27	DSM 30,104	*K. pneumoniae* (hypermucoid strain)	DSMZ Culture Collection	ERS29671980
28	KR311	*K. pneumoniae*	Leicester Royal Infirmary	ERS29686510
29	MH05	*K. pneumoniae*	Leicester Royal Infirmary	ERS29686511
30	Top52	*K. pneumoniae*	Scott Hultgren’s Collection (UTIs patient) (33, 34)	ERS29686512

^*a*^ Full information on the strains in Table S1.

Comparison of 311F/05F and Leicester-UTI1 (Fig. 1b, *P < 0.05) revealed that the addition of four new phages (Centimanus, Storm, Anoxic, and SoFaint) significantly improved the coverage. Leicester-UTI1 demonstrated greater breadth of activity, targeting more strains than 311F/05F. A closer examination of individual bacterial strains suggests that the addition of phages is strain dependent. A synergistic effect was observed in most of the bacterial strains; however, an antagonistic interaction was also identified, particularly in *K. pneumoniae* KR311, 13440, ARGID 36294, M201312995Q, and M201353511C. The addition of four-phages (Centimanus, Storm, Anoxic, and SoFaint) to 311F/05F appeared to reduce the killing efficacy against these strains, although the local *vi* score still indicated a medium killing activity (*vi* 0.20–0.50). The observed synergistic and antagonistic effects highlight that the growth and infection dynamics of one phage can influence those of others within the same bacterial host. Antagonistic effects or interference imply that one or more phages negatively impact the efficacy of others, potentially due to receptor competition or interference of lytic cycles (35). In contrast, synergistic interactions indicated enhanced key lytic phage characteristics, such as adsorption rate (infection efficiency), burst size (number of progeny produced), and lysis time (duration from infection to progeny release) (36).

Previous genomic analysis conducted by Jameson et al. (27) provided additional insights, confirming that phage Centimanus in Leicester-UT1 encodes nine depolymerase proteins. These enzymes disassemble bacterial capsules, thereby facilitating phage infection (36, 37). While these findings highlight the potential of Centimanus in enhancing cocktail efficacy, further research is necessary to elucidate the detailed mechanisms of its depolymerase activity (38).

Murine models have been instrumental in elucidating UTI pathogenesis but have rarely been adapted to study how phage dynamics within a complex bladder environment. Unlike other rodent models, mice do not naturally exhibit vesicoureteral reflux, which can lead to UTIs (39). However, they share physiological similarities with humans, such as a comparable proportion of globoseries glycolipid receptors on urothelial cells, which facilitate bacterial attachment (40), and conserved uroplakins that promote type 1 fimbriae adherence and uropathogenic invasion (41). Experimentally, several instillation techniques, including unobstructed UTI instillation and transurethral catheterization, have been developed to precisely induce controlled and reproducible infections (42).

In this study, we used the *K. pneumoniae* Top52 strain, a clinical isolate from a female patient with recurrent UTIs that has previously been used in a murine pneumonia model (33, 43). Encouragingly, within our C57BL/6J model, persistent high bacterial titers (>10^4^ CFU/mL) were maintained over 7 days, mimicking acute human bladder infection (44). Selecting an appropriate bacterial strain is critical to establishing murine UTI models, as phenotypic differences in bacterial behavior can arise between human and mouse environments due to variations in development and growth conditions (45). Additionally, bacterial virulence factors also dictate behavior; therefore, the selected strains should exhibit similar pathologies that closely model the UTIs (1).

One of the problems that makes UTI bacteria so problematic is that they can form intracellular bacterial communities (IBCs) (46). Upon invading bladder epithelial cells, these bacteria proliferate within the cytoplasm and expand into the bladder lumen as colonies grow (46). These colonies can form biofilm-like structures, invade deeper urothelial layers, and persist in membrane-bound compartments with limited metabolic activity—a state known as “quiescent intracellular reservoirs.” These reservoirs remain latent but can reactivate when the upper urothelium exfoliates (47, 48).

Our results show that although the *in vitro vi* for Leicester-UTI1 against *K. pneumoniae* Top52 was 0.35 (classified as medium) (26), complete clearance of bacterial burden in the urine and bladder was achieved by day 7. This is consistent with work by Singh et al. (24), which demonstrated the efficacy of transurethrally administered phage therapy in a murine UTI model. However, our findings suggest that the Leicester-UTI1 cocktail exhibits enhanced activity *in vivo* relative to *in vitro* predictions.

Importantly, our data extend beyond urine-based assessments, indicating that phage therapy may also impact tissue-associated infection, including IBCs within the bladder. We hypothesize that the observed clearance reflects both localized phage replication within the urinary tract and interactions with the host immune response. By reducing bacterial burden, phages may facilitate immune-mediated clearance of residual populations (49). In addition, phage binding to bacterial surfaces has been shown to enhance phagocytosis by immune cells, potentially increasing bacterial susceptibility to host defense mechanisms (50, 51). Together, these observations highlight the importance of considering phage–host interactions when evaluating therapeutic efficacy.

Phage recovery and dynamics analysis further reveal that the phage was able to actively replicate in the urinary lumen in which the bacteria were present. However, comparable phage titers were observed in the bladder between PHAGE and KLEB-PHAGE groups (Fig. 2c), indicating limited intracellular phage replication. This is consistent with previous literature depicting that host defense mechanisms may limit phage multiplication intracellularly and thus prevent phage systemic circulation (52). Furthermore, no phages were detected in the blood or spleen, also suggesting that phage activity remains localized.

A histopathological analysis (Fig. 3a through c) revealed that *K. pneumoniae* Top52 formed IBCs, invaded urothelial cells, and triggered inflammation extending into the smooth muscle. In contrast, phage treatment appeared to protect against severe inflammation and potentially facilitated tissue recovery. This effect may, of course, result from the reduced bacterial burden achieved through phage therapy, but our data suggest that the picture is more complex. Indeed, phages have been shown to elicit diverse immune responses in humans (42). For instance, Pf4 phage treatment for *Pseudomonas aeruginosa* infections has been shown to induce IFN-β and downregulate pro-inflammatory TNF (53). Upregulation of anti-inflammatory cytokines after phage application has also been observed in a murine study, which showed injected phages induced systemic IFN-β, providing additional protection against vaccinia virus infections (54). Of critical relevance to this study is that phages have been reported to induce IL-10 expression, which is an anti-inflammatory and regulatory cytokine that mitigates tissue damage (55, 56). The *in vitro* finding of phage application effect on monocytes is consistent with our observations (55). In our study, the mice that received phage therapy had upregulation of systemic and localize IL-10 (Fig. 4a and b). IL-10 has been extensively studied as a major tissue-protective cytokine, which can protect the host against Th1- and Th2-associated tissue damage exerted by bacterial infections (56). This explains the lower inflammation score in the bladder tissue of phage-treated mice compared to the infection control, as phages induced IL-10 and suppressed tissue inflammation. Although IL-10 production often coincides with IL-6 expression—a critical inflammatory cytokine involved in viral resolution and Th2 cell expression—which is also essential to get a balanced immune response (53, 57, 58).

While this study highlights the potential of phage therapy as a treatment for UTIs, it is important to acknowledge the study limitations. The limitations include the volume of urine retrieved from mice, which could not be standardized, as not all mice produced daily urine samples. Efforts were made to address this using metabolic cages and positive reinforcement with treats, yet occasional failures to collect samples persisted. Second, the relatively short study duration of 7 days means that understanding treatment for chronic infections lasting more than 2 weeks or recurrent UTIs is limited. Therefore, running longer experiments might be necessary to test the phage for chronic UTIs treatment. Additionally, assessing adaptive immune responses, such as antibody (e.g., IgA and IgG) production during chronic infection and longer phage exposure, would be valuable to investigate further host responses to phage therapy (53).

In summary, we showed for the first time that we have a system to explore the efficacy of phages in a robust preclinical model, which has allowed us to gather important insights into how phages could be exploited to treat UTIs in humans. The robust data set demonstrates that, for uncomplicated acute UTIs, a single dose of the phage cocktail is sufficient to remove the bacterial pathogen and resolve the infection. Furthermore, the observed anti-inflammatory response exerted in the phage-treated group through transurethral injection underscores the potential applicability of phage cocktails in reducing inflammation caused by UTIs. The histopathology data also support the anti-inflammatory effects of phage therapy, which is critical to solving the infection.

In future work, the cocktail could be expanded to address polymicrobial infections, for example, by incorporating additional phages targeting other bacterial species. For instance, prior research by Haines et al. (26) combined *E. coli* and *Klebsiella* phages, demonstrating efficacy toward both species within the mixed cocktail. Additionally, future research on the use of phage-antibiotic combinations *in vivo* will be essential, as phages are often used in conjunction with antibiotics rather than as standalone therapies (59). Notably, therapeutic synergy between antibiotics and phages has been shown to improve patient outcomes in cases where phages have been used under compassionate grounds (60–63).

## MATERIALS AND METHODS

### Strains and phages used in this study

Six phages and their hosts were used in this study and referred to as the Leicester-UTI1 cocktail. They were sourced from the Becky Mayer Center for Phage Research (Table 1) (Information S1). A panel of 30 *Klebsiella* strains was used in this study; 29 were ESBL-producing *Klebsiella* strains isolated from patients with UTIs sourced across multiple hospitals and were used to test phage host range (Table 2). The final strain was Top52 #1721 (Top52), sourced from Scott J. Hulgren and used *in* the *in vivo* mouse model (33). All strains were cultured from frozen stocks on LB agar medium (1.5% [wt/vol], Luria-Bertani media Sigma-Aldrich, United Kingdom L3522, Bacteriological Agar, VWR, United Kingdom), supplemented with 1 mM CaCl_2_ (Fisher Chemical, Fisher Scientific, United Kingdom) and incubated at 37°C overnight (~18 h). The liquid culture was grown in LB medium and incubated overnight at 37°C in a shaking incubator at 300 rpm.

### DNA extraction and oxford nanopore sequencing

Genomic bacterial DNA (except *K. pneumoniae* 13,440 and 30,104 genomes, as they were already available) was extracted from an overnight (16 h) liquid culture (1.5 mL) using the DNeasy Blood and Tissue kit (QIAGEN) following the manufacturer’s protocol for gram-negative extraction and processed on a QIAcube Connect (QIAGEN) automated extraction platform. The DNA concentration was quantified using an Invitrogen Qubit 4 fluorometer (Invitrogen), following the manufacturer’s instructions for the Qubit 1 × dsDNA broad range sensitivity assay kit (Invitrogen).

Sequencing libraries were prepared using Rapid sequencing DNA V14 Barcoding Kit (SQK-RBK114.24) and sequenced in-house via Oxford Nanopore Technologies on a GridION using R10.4.1 flow cells. Base calling was carried out using the high-accuracy v4.3.0 400 bps model, and subsequent trimming of barcodes and adapter sequences was performed with MinKnow.

### Genome assembly, annotation, and phylogeny

Genome assemblies were generated using Autocycler v0.5.2 with the fully automated assembly parameters (64). Assembly quality metrics were assessed using CheckM2 v1.1.0 (65). Genome annotation was performed using Bakta V1.12*. Klebsiella-specific genomic annotations were carried out using Kleborate v3.2.4 (66, 67). Antimicrobial resistance determinants were identified using RGI v6.0.5 from CARD v4.0.1 with selection criteria of “strict” or “perfect” hits only (44). Phylogenetic reconstruction was performed using GTDB-Tk v2.1.1 with the GTDB release 226 (45). A curated set of 120 conserved bacterial marker genes was identified and aligned using the *de novo* workflow (gtdbtk de_novo_wf). *Staphylococcus casei* DSM 15,096 was included as an outgroup. A maximum-likelihood phylogeny was constructed that was visualized and annotated using iTOL v7 (68).

Additionally, the six phages used in this study had been isolated previously, and there was *a priori* information about their taxonomy (26, 27). For each of the known families the phages had been classified into, at least two representatives’ genomes from each genus in a family were used as input to VipTreeGen (69). Trees were visualized with iTOL (version 6.0) (68).

### Phage propagation, titration, and purification

The double-layer agar method was used for each phage propagation from the stock culture. In brief, bacterial strains were cultured to log phase (OD_600_ = 0.15–0.2) using an overnight culture as a starting point. A total of 200 μL of bacteria culture and 200 μL of phage stock were then added to 8 mL of 0.7% (wt/vol) LB agar, poured onto square LB 1.5% (wt/vol) agar plates, and incubated at 37°C overnight (~18 h). The next day, the top layer agar was scraped and transferred into a 50 mL Centrifuge Tube with 10 mL SM Buffer (100 mM NaCl [Sigma-Aldrich, United Kingdom], 8 mM MgSO4•7H_2_O [Sigma-Aldrich, United Kingdom], and 50 mM Tris-HCl pH 7.5 [Sigma-Aldrich, United Kingdom]) and incubated at room temperature for ~18 h. The phage lysate was centrifuged at 4,000 × *g* for 15 min, and the supernatant was filter-sterilized through 0.2 μm pore size filters. The phage stock titer was determined using plaque assays. The phage stocks were stored at 4°C.

### Phage host range with planktonic killing assay

To determine the host range of the cocktail, a PKA was performed using a previously described method (26). This optimization step was the development of the previously described *Klebsiella* phage cocktail: 311F and 05F. We then developed further by testing the addition of phages Anoxic, SoFaint, Centimanus, and Storm into the cocktail (Leicester-UTI1) with the ratio of 1:1:1:1:1:1. Briefly, bacteria overnight cultures were diluted 1:100 and added to flat-bottom 96-well plates in triplicate per strain. Negative controls used LB only and Blanks used LB with Gentamicin 2 μg/mL. The bacteria were grown to OD600 0.15 (1 × 10^8^ CFU/mL), and 100 μL of phage cocktail (containing 10^8^ PFU/mL of each individual phage) was added to each well, and dilutions of phages were made using LB media. The PKA was done at an MOI of 1. The experiments were performed using the BMG Labtech SPECTROstar Omega. The program was set up as follows: OD600 taken every 5 min for 8 h with shaking 10 s before each reading. The result was merged using the program to produce a killing assay curve. The curve was then analyzed by a method developed by Storms et al. (31). The local *vi* score was a ratio of the area under the bacteria growth curve challenged with phage cocktails against the bacteria growth curve without phage (positive control) during the log phase. The local *vi* was produced using Rstudio (https://www.R-project.org/) (Information S1). The local *virulence index* gives a value between 0 and 1; 0 means not effective and 1 means highly effective. The local *vi* score was categorized into low (local *vi* < 0.2), medium (0.2 ≤ local *vi* ≥ 0.5), and high (local *vi* > 0.5). An effective killing is described as having a medium and high local *vi* score.

### Phage endotoxin purification

Endotoxin purification is required for phage products to fulfill the minimal standard for a clinical product. For intravenous application, the limit is 5 EU/mL, and for oral administration, it is <20 EU/mL. Endotoxin purification was carried out using commercial endotoxin affinity assay Endotrap HD (LIONEX, GmBH) following the manufacturer’s instructions. In brief, 1 mL of purified phage in SM-Buffer was run through the column using a gravity column. A total of 200 μL of void volume was collected in a separate tube. The total phage lysate volume run through was 20 mL. Endotoxin quantification level was performed using Pierce Chromogenic LAL Assay (ThermoFisher, United Kingdom) as per its instructions. The purified Leicester-UTI1 lysate was shown to retain the same killing ability as the unpurified version (Fig. S1).

### Experiment design of infection model *in vivo*

For the *in vivo* study, the method was modeled according to the well-established protocol (28). *K. pneumoniae* Top52 was cultured in LB medium and incubated statically for 18–24 h at 37°C, then sub-cultured 20 μL into 20 mL fresh LB broth and incubated statically again for 18 h at 37°C. The culture was centrifuged at 4,200 RPM for 10 min, and the pellet was resuspended in 10 mL sterile PBS (Sigma Aldrich, United Kingdom). The OD600 was normalized to 0.91–1.00, which corresponds to approximately 2–4 × 10^8^ CFU/mL (1–2 × 10^7^ CFU in 50 μL). The titer was verified using serial dilutions and the spotting method on cystine-lactose-electrolyte-deficient (CLED) agar medium (VWR, No. 75,813, USA)

Female C57Bl/6J mice aged 7–8 weeks were bred and maintained in-house at the University of Leicester. Mice were caged in a room with a 12/12 h light-dark cycle and had *ad libitum* access to water and food pellets. In the experiment, mice were randomized into four groups: group 1 PBS CONTROL (*n* = 9) with PBS at 0 h and SM-Buffer at 48 h, group 2 PHAGE (*n* = 17) with PBS at 0 h and 10^6^ Phage cocktail at 48 h, group 3 KLEB (*n* = 15) with *K. pneumoniae* Top52 at 0 h and SM-Buffer at 48 h, and group 4 KLEB-PHAGE (*n* = 23) with *K. pneumoniae* Top52 at 0 h and 10^6^ PFU in a total volume of 50 μL PHAGE cocktail at 48 h (Fig. 2a). Mice were lightly anesthetized with 2.5% (vol/vol) isoflurane over oxygen (1.8–2 L/min), and the procedure was done via transurethral injection with 50 μL volume. At each timepoint, mice were humanely killed by terminal anesthesia and immediately dissected. Bacterial levels at day 0, day 2 (4 h after intervention, either phage treatment or PBS), and day 7 were defined as the abundance of *K. pneumoniae* Top52 per mL in bladder, kidneys, and spleen tissue lysate. Urine samples were attempted to be collected from all mice every day starting from day 1 up to day 7. Positive reward training or bladder massaging methods were used to encourage the mice to urinate on the petri dish. Alternatively, the mice were taken into metabolism cages for 1–3 h until they produced urine. The bladder, kidneys, and spleen were homogenized using FastPrep Homogenization (FastPrep 24). The homogenized organs were then serially diluted and plated on CLED agar plates for bacterial enumeration. The rest of the homogenized solutions were processed for phage counting by using the plaque assay method on double-layer agar as mentioned above. Blood samples were also taken via cardiac puncture during the terminal anesthesia procedure, and samples were processed for counting bacterial load. Additional mice were used for histopathology analysis following the same infection model.

### Histopathology

The bladders and kidneys of the mice were reserved for histopathology assessment in 10% formalin (VWR International, United Kingdom). Organ sections of 5 μm thick were cut from paraffin-embedded (Leica HistoCore Pearl Tissue Processor) and stained with hematoxylin and eosin. The histopathological grading scale for the degree of inflammation (0–5) was adapted from the original definition by Hopkins (41), with additional detail changes presented in Table 1. Slides from the CONTROL group (untreated mice) were used as a baseline for uninfected tissue. A blinding method was applied during the grading process, so that the treatment groups and culling time points were kept confidential to limit reporting bias.

### Phage susceptibility testing of *Klebsiella* colonies post-murine infection

*Klebsiella pneumoniae* Top52 colonies (yellow colored) were isolated from bladder samples of group 2 KLEB-PHAGE on day 7. Colonies were streaked on CLED agar plates and incubated overnight at 37°C. The colonies were re-streaked two times on CLED agar plates. The plates were then stored at 4°C prior to phage sensitivity testing. Recovered colonies were inoculated in LB broth and incubated overnight at 37°C in a 300 rpm shaking incubator. Sensitivity of recovered colonies was tested against Leicester-UTI1 (311F, 05F, Anoxic, SoFaint, Centimanus, and Storm) with 10^8^ PFU/mL concentration (MOI of 1) using PKA method. The local virulence index was calculated as previously explained. The growth and doubling times of recovered bacteria were analyzed using the Dashing Growth Curve (70) (Table S2).

### IL-6, IL-10, TNF-alpha, and IFN-gamma expression

The serum was retained from mouse blood by centrifuging the fresh blood at 4°C at 9,000 RPM and was stored at −20°C. The bladder lysate was kept at −20°C and centrifuged before the enzyme-linked immunosorbent assay (ELISA) was performed. The bladder samples were diluted 10× or 100× before the experiment. The ELISA was performed using commercially available kits (BioLegend; IL-6, Cat. no. 431304; IL-10, Cat. no. 431414; TNF-alpha, Cat. no. 430904; IFN-gamma, Cat. no. 430804) as per the instructions. All samples were done in triplicate.

### Statistics

Graphic and statistical analyses were performed using GraphPad Prism 7 (GraphPad Software, Inc). For both phage cocktail comparison and animal experiments with two groups, the variances of the outcome variables were compared to select the appropriate statistical tests. Shapiro-Wilk test was performed to verify normal distribution. A two-tailed *t*-test was used if the data were normally distributed, or a two-tailed Mann-Whitney *U* test if not. All *in vitro* experiments were performed in triplicate.

## Supplementary Material

Reviewer comments

## SUPPLEMENTAL MATERIAL

Supplemental material spectrum.03952-25-s0001.docx Fig. S1 to S5, Data S1 and S2, and script used to analyze local virulence index.

Graphical abstract spectrum.03952-25-s0002.tiff Visual summary of the study.

Tables S1 and S2 spectrum.03952-25-s0003.xlsx Host information and antibiotic resistance profiling.

Tables S3 and S4 spectrum.03952-25-s0004.xlsx Host range and growth curve analysis.

OPEN PEER REVIEW reviewer-comments.pdf An accounting of the reviewer comments and feedback.

ASM does not own the copyrights to Supplemental Material that may be linked to, or accessed through, an article. The authors have granted ASM a non-exclusive, world-wide license to publish the Supplemental Material files. Please contact the corresponding author directly for reuse.
