# Supplementary material for: Development and in-vivo efficacy of Leicester-UTI1 phage cocktail that targets ESBL-associated Klebsiella from urinary tract infections
Source: Microbiol Spectr. 2026 Jun 24;14(8):e03952-25. doi: 10.1128/spectrum.03952-25 (PMC13436347; doi:10.1128/spectrum.03952-25)
Supplement: Supplementary file 1 [file reviewer-comments.pdf]

AMERICAN SOCIETY FOR MICROBIOLOGY | Microbiology Spectrum

Development and *in-vivo* efficacy of LEICESTER-UTI1 phage cocktail that targets ESBL-associated *Klebsiella* from urinary tract infections

Rizka Jariah, Jennifer Schofield, Kevin West, Karen Adler, Slawomir Michniewski, Caitlin Wildsmith, Eleanor Jameson, Lucy Onions, Faizal Patel, Anusha Saravanan, Hasan Yesilkaya, Spyridon Megremis, Andrew Millard, Martha Clokie, and Melissa Haines

Corresponding Author(s): Martha Clokie, University of Leicester

Review Timeline:

Submission Date:	December 6, 2025
Editorial Decision:	February 20, 2026
Revision Received:	April 22, 2026
Accepted:	May 21, 2026

Editor: David Pride

Reviewer(s): Disclosure of reviewer identity is with reference to reviewer comments included in decision letter(s). The following individuals involved in review of your submission have agreed to reveal their identity: Frank Oechslin (Reviewer #3)

Transaction Report:

(Note: With the exception of the correction of typographical or spelling errors that could be a source of ambiguity, letters and reports are not edited. The original formatting of letters and referee reports may not be reflected in this compilation.)

DOI: https://doi.org/10.1128/spectrum.03952-25

Re: Spectrum03952-25 (Development and in-vivo efficacy of LEICESTER-UTI1 phage cocktail that targets ESBL-producing Klebsiella species in urinary tract infections)

Dear Dr. Martha R.J. Clokie:

Thank you for the privilege of reviewing your work. Below you will find my comments, instructions from the Spectrum editorial office, and the reviewer comments.

Please return the manuscript within 60 days; if you cannot complete the modification within this time period, please contact me. If you do not wish to modify the manuscript and prefer to submit it to another journal, notify me immediately so that the manuscript may be formally withdrawn from consideration by Spectrum.

Revision Guidelines
To submit your modified manuscript, log into the submission site at https://spectrum.msubmit.net/cgi-bin/main.plex. Go to Author Tasks and click the appropriate manuscript title to begin. The information you entered when you first submitted the paper will be displayed; update this as necessary. Note the following requirements:

• Upload point-by-point responses to the issues raised by the reviewers in a file named "Response to Reviewers," NOT in your cover letter.
• Upload a compare copy of the manuscript (without figures) as a "Marked-Up Manuscript" file.
• Upload a clean .DOC/.DOCX version of the revised manuscript and remove the previous version.
• Each figure must be uploaded as a separate, editable, high-resolution file (TIFF or EPS preferred), and any multipanel figures must be assembled into one file.
• Any supplemental material intended for posting by ASM should be uploaded with their legends separate from the main manuscript. You can combine all supplemental material into one file (preferred) or split it into a maximum of 10 files with all associated legends included.

For complete guidelines on revision requirements, see our Submission and Review Process webpage. Submission of a paper that does not conform to guidelines may delay acceptance of your manuscript.

Data availability: ASM policy requires that data be available to the public upon online posting of the article, so please verify all links to sequence records, if present, and make sure that each number retrieves the full record of the data. If a new accession number is not linked or a link is broken, provide Spectrum production staff with the correct URL for the record. If the accession numbers for new data are not publicly accessible before the expected online posting of the article, publication may be delayed; please contact production staff (Spectrum@asmusa.org) immediately with the expected release date.

Publication Fees: For information on publication fees and which article types are subject to charges, visit our website. If your manuscript is accepted for publication and any fees apply, you will be contacted separately about payment during the production process; please follow the instructions in that e-mail. Arrangements for payment must be made before your article is published.

ASM Membership: Corresponding authors may join or renew ASM membership to obtain discounts on publication fees. Need to upgrade your membership level? Please contact Customer Service at Service@asmusa.org.

The ASM Journals program strives for constant improvement in our submission and publication process. Please tell us how we can improve your experience by taking this quick Author Survey.

Thank you for submitting your paper to Spectrum.

Sincerely,
David Pride
Editor
Microbiology Spectrum

Reviewer #2 (Comments for the Author):

Major comments:
I agree that this is very important work and supplies much needed data on phage therapy of Klebsiella UTIs, but some claims are over-stated and/or misleading and the paper can benefit from simplified visualizations, removal of ghost text from previous version(s) and tightening up of the text.

My detailed review is attached.

Reviewer #3 (Comments for the Author):

This manuscript presents a rigorous evaluation of the Leicester-UTI1 phage cocktail as a therapeutic strategy against uropathogenic Klebsiella pneumoniae, including ESBL-producing clinical isolates. The authors demonstrate strong in vitro efficacy across a clinically relevant panel and validate these findings in a mouse model that closely mimics acute urinary tract infection. Phage treatment significantly reduced bacterial burden in urine, bladder, and kidney within hours of administration, without evidence of resistance emergence. The additional analysis of inflammatory markers further strengthens the translational relevance of the work. The data are compelling,, and the conclusions are well supported.

Only minor comment is that the text within several figures appears somewhat small and difficult to read.

Overall, this is an excellent and clinically relevant study that makes a meaningful contribution to the field of phage therapy.

Major concerns:

1) The number of panel strains tested seems to be rather low, only assessing 30 strains. Reference 23 (Singh et al, 2024) tested a panel of 70 strains in their UTI phage therapy study and other groups typically use 100-strain panels. This is to ensure that the phage cocktail can handle sufficient host diversity. It is difficult to tell the level of panel diversity here as no genomes are available, not phylogeny was done and not every strain in Table 3 has data for K and O antigens.

2) The apparent lack of phage clearing of the in vitro cultures shown in Figure 1d is concerning as the phages don't seem to be doing anything until very late into log phase. What do these plaques look like if the culture gets and remains that dense? EOP data work give more confidence that these phages are robust enough for phage therapy. Judging from the PKA or growth curve data presented in Fig 1d, it seems to this reviewer that the in vitro efficacy of 77% is inflated and that the real value is closer to 48% (15/31) to 61% (19/31). Many of these lines remain flat and probably should not be counted as they fail to clear

3) While the 6-phage cocktail did have activity on more strains than the 2-phage cocktail, there does seem to be evidence of antagonism that was not discussed (e.g., Fig 1d strains 13440 and KR311 showed that the 2-phage cocktail worked better than the 6-phage cocktail).

4) The claim that no resistance to phage occurs in vivo is misleading because only 2 mice were tested using a Kp strain from a recurrent UTI patient which means this strain can hide out intracellularly, increasing the likelihood that they escape from the phages altogether. It would be nice to see EM images of bladder cells to assess where the Kleb cells are hanging out (inside cells?). It is not clear why many more samples were not tested for resistance or why spot testing was not performed to evaluate resistance. Why not test every sample for resistance? At least test Urine on day 5, which showed an uptick in bacterial growth from day 4.

5) The figures are overly complex making it difficult to read and distracting the reader from the main points. The figure layout in the biorxv is much better.
- For Fig 1, I suggest removing 1a as this is not needed because this information is in the methods section. 1c can be removed or placed into supplemental data. That leaves 1b and d. Both can be made larger and the x-axis labels can be added to 1d (we need to see each hour of the growth curve).
- Fig 2a can be moved to supplemental. Also, it is not clear what the efficacy figure is supposed to be or the chart under inflammation as they are not described in the legend. 2b: The photo of the mice is not necessary and the rest of 2b can be moved to supplemental because this mouse model is not new. All that is needed in Fig 2 is 2c and d. It would be helpful to see where the phage was added in 2C (maybe with an arrow and a Ò?Ó symbol). 2e can also be moved to supplemental as it only shows that phage can infect the 3 recovered strains from 2 mice, and not well either.

- Fig 3 should be split into 2 figures: 3a and b and then c would be figure 4. The bargraphs are just too small and the text on the x-axis is not ledgable. Also, the red color in the 3a right plot looks too brown. It needs to be the same colour as the legend.
- I suggest combining Table1 content into Figure 3a+b as a key for the grading scale and remove Table 1.
- Table 2 is ok
- Table 3 needs to have data for every K and O locus. Presumbably Kaptive was used to determine K and O loci? That is not mentioned anywhere in the paper! Any bacterial genomics and bioinformatics that was used needs to have text in the methods section. I also want to see the ESBL bla phenotypes or genotypes in Table 3 so the reader can determine that they are indeed ESBL and how diverse their AMR profiles are.

Minor comments (line by line):

1) lines 76,77: Add references to case reports using phage to treat Kp UTIs

2) When citing reference 23, add more information about what that study provided Ð it is currently glossed over and insignificant. They used a 3-phage cocktail tested on a 70-strain panel of UTI clinical Kp strains. They also tested different MOIs and inoculation methods/routes which set the stage for your study (i.e., you didnÕt have to test MOI or inoculation route). Another distinction is that they inoculated with phage every day.

3) line 92: italicize Òin vitroÓ

4) line 94,94-112: change ÒobligatoryÓ to ÒobligateÓ and make sure that the order of figure references matches the order presented in the text. Currently Fig 1c is mentioned before b, before a.

5) line 101: move Figure 1a to supplemental or remove entirely

6) line 140,141: move Figure 2b to supplemental

7) line 144: ÒCFU/mlÓ should be ÒCFU/gÓ as that was how you reported it in Fig 2d for bladder.

8) lines 148-150: Make the order of your experimental groups be the same as Fig2 and the Methods (Methods order is different).

9) lines 163-170: To help the reader be confident that the implementation of this mouse model reflects human UTIs as much as possible, it would be useful to report any clinical symptoms that the mice had (e.g., fever, increase wbc, etc)? How do you know they have a UTI vs. asymptomatic bacteruria?

10) line 172: change ÒlevelsÓ to ÒabundanceÓ

11) line 177: change Òin both infected groupsÓ to Òin both KLEB (or Klebsiella-infected) groupsÓ if that is what you mean here. Remember,

you are ÒinfectingÓ with 2 different things (bacteria and phage).

12) lines 180-183: I really like this result and this needs to be discussed in the Discussion section. Singh et al used daily phage treatments in their study whereas you used only one. That is super important to know in the clinic. Also testing where the phage goes (bladder, kidney) and does not go (blood or spleen) is pharmacokinetics and this needs to be played-up more in the Discussion and also in the abstract if room. Currently this important point is buried in the text.

13) lines 193-201: This was mentioned in the Major concerns section of the review. More samples need to be tested (urine and bladder). Also include spot plaque assays.

14) Legend for Fig 3. State what **, ***, **** represent. Are they different P-values?

15) line 220: I think you want to say ÒPBS CONTROL groupÓ served as a baseline as it doesnÕt make much sense to use the Klebsiella Control group (Fig 3a key) for baseline. Alternatively, you can clarify the key to Fig 3a with ÒKLEBÓ in red, ÒKLEB-PHAGEÓ in blue and ÒPBS ControlÓ in green. Consistency of group labels across figures will make this read better.

16) line 243: ÒIFN-gamma, wereÓ to ÒIFN-gamma wereÓ

17) lines 239-262: Order of presentation. It would be easier to follow with Fig 3c (new Fig4) if you presented the bladder results first and then serum. Alternatively, just change the order in Fig 3c to match the order presented in the text.

18) line 269,270: Establishing a mouse model does not address the gap as multiple mouse models exist (e.g., ref 26 and 23 and others not cited). The gap being addressed is testing the efficacy of the improved 6-phage cocktail in vivo.

19) line 294,295: Òtarget as many as possible clinical strainsÓ to Òtarget as many clinical strains as possibleÓ.

20) line 302: add Òin vivoÓ after Òbacterial growth.Ó

21) line 303: please clarify the synergy statement since 13440 and KR311 both seemed to have antagonism in the 6-phage cocktail compared to the 2-phage cocktail.

22) lines 307-312: ÒIndeed, genomic analysis (Table 3)ÉÓ This seems to be ghost text from a previous version perhaps as no genomic analysis was mentioned in the Methods or Results sections and no depolymerase data is presented in Table 3. Either removed this text or add the data to the manuscript.

23) line 325: Òin murineÓ to Òin a murineÓ

24) line 327: Òmimicking acute human bladder infectionÓ As far as I can

tell, no clinical data (e.g. body temp/fever, white blood cell count, etc) was presented to suggest nothing more than asymptomatic bacteriuria.

25) line 328: I would argue that this strain Top52 might not be the best for strain for acute UTI as it seems to be more appropriate in a model of recurrent UTI since it came from a patient with recurrent UTI and you presented supporting data that it can hide out in the bladder, presumably escaping phage attack. Again, an EM of these cells would show Kleb inside cells.

26) line 366,367: Perhaps discuss determining which of the 6 phages cause IL-10 induction? Do you see that with the 2-phage cocktail.

27) It would be intellectually useful to discuss 2 vs 3 vs 6-phage cocktail in a paragraph also discussing the difference between Singh et al and this study. Multiple doses are not required for the 6-phage cocktail here but maybe for 2 or 3-phage cocktails? These are important factors to consider when developing a therapeutic phage cocktail.

28) under limitations: Please add that the adaptive immune response was not evaluated (e.g., IgA, IgG). Only 2 mice and 3 colonies of recovered Kleb were evaluated for phage resistance. Only 30 strains were tested in vitro and that an expanded panel needs to be screened to determine the effectiveness of the 6-phage cocktail in the clinic. It would be useful to have host range data of each phage independently as supplemental data to determine if all 6 phages are required.

29) line 415: Luria Bertani is the incorrect name according to Bertani, 2004 (PMC321500). It is supposed to be called ÒLysogeny BrothÓ. If the authors feel strongly to keep Luria Bertani, I suggest hyphenating it as ÒLuria-BertaniÓ.

30) line 429: please italicize Òa prioriÓ

31) line: 441: ÒmmMgSO4Ó should be Òmm MgSO4Ó

32) Phage host range PKA section. Was each phage added in equal amounts to the cocktail in a ratio of 1:1:1:1:1:1?

33) lines 492-495: please make sure the group number order matches the text and Figs.

34) line 526: Were yellow colonies picked? On CLED, yellow colonies should be Klebsiella.

35) line 529: ÒCocktail 2Ó should be ÒLEICESTER-UTI1Ó no?

36) line 530: ÒMOI 1Ó to ÒMOI of 1Ó

37) line 532: ÒDASHÓ should be ÒDashing Growth CurvesÓ according to https://dashing-growth-curves.ethz.ch/

38) Phage susceptibility testing post murine infection. Please state at

what timepoint and how many mice were tested (2 according to the text). Also, did you really test urine samples for phage resistance? If so, which timepoint(s)?

39) references 10 and 11 need to have a space after 10. And 11.

40) references: please italicize all bacteria genus and species names. Ref 23 for sure needs Klebsiella pneumoniae to be in italics.

41) Are any of the panel strains hypermucoid?

42) Do you observe any phage resistance in vitro? Kp is notorious for rapidly obtaining phage resistance.

43) I recommend changing the title by removing ÒLEICESTER-UT1 phageÓ to Òa 6-phageÓ

We appreciate the reviewer's careful reading of the manuscript and constructive feedback. We have focused on tightening the text, clarifying areas where claims could be misinterpreted, simplifying figures, and removing residual text from earlier versions. We addressed point by points below:

Reviewer 2

Major comments:

I agree that this is very important work and supplies much needed data on phage therapy of Klebsiella UTIs, but some claims are over-stated and/or misleading and the paper can benefit from simplified visualizations, removal of ghost text from previous version(s) and tightening up of the text.

My detailed review is attached.

1. The number of panel strains tested seems to be rather low, only assessing 30 strains. Reference 23 (Singh et al, 2024) tested a panel of 70 strains in their UTI phage therapy study and other groups typically use 100-strain panels. This is to ensure that **the phage cocktail can handle sufficient host diversity**. It is difficult to tell the level of panel diversity here as no genomes are available, not phylogeny was done and not every strain in Table 3 has data for K and O antigens.

   We agree that panel diversity is critical. The 30 strains included here were selected to represent clinically relevant diversity across UK collections. To make this clearer, we have now strengthened the genomic characterisation presented. Specifically, we have added expanded information on sequence types (ST), K- and O-loci, and resistance profiles (Table S1-S2), and included a phylogenetic analysis (Figure S1a). CARD-based analysis is also available (Table S2; Figure S1b). We have also clarified this in the Result section **'Expanding phage cocktail breadth against diverse UTI-associated Klebsiella isolates'**

   A set of 30 strains UTI-associated and clinically relevant ESBL and non-ESBL Klebsiella from diverse UK sources was used in this study (Table 2). These comprised of five species, predominantly K. pneumoniae (26/30) with the rest being K. michiganensis, K. oxytoca, K. variicola, and K. quasipneumoniae (Figure S1a) (Table S1). The maximum likelihood tree showed that all isolates clustered according to their assigned species, with internal node support values >70% across the majority of branches (Figure S1a). Sequence types (ST) of analysis confirms the diversity of the strains, with 22 distinct ST-types were identified (Table S1). Among these, ST147 – associated with hypervirulence and resistance plasmid (25) were observed in Leicester isolate (KR397) and Bristol isolate (P2.14) (Table S1). Capsule typing further revealed the diversity with distinct 25 K-loci and 7 O-loci (Table S1), indicating surface antigen variability. Genomic AMR predictions using CARD identified a total of 89 AMR determinants across the isolates (Figure S1b) (Table S2), with 40% (12/30) observed to carry ESBL (bla) genes, 36.7% (11/30) were carbapenems producer, and 5 strains harboured both (Table S1). This heterogeneity highlights the urgency for phage cocktails with broad host range. To address this, the Leicester-UTI1 phage cocktail (Table 1) was developed through a stepwise optimisation process.

2. The apparent lack of phage clearing of the in vitro cultures shown in Figure 1d is concerning as the phages don't seem to be doing anything until very late into log phase. What do these plaques look like if the culture gets and remains that dense? EOP data work gives more confidence that these phages are robust enough for phage therapy. Judging from the PKA or growth curve data presented in Fig 1d, it seems to this reviewer that the in vitro efficacy of 77% is inflated and that the real value is closer to 48% (15/31) to 61% (19/31). Many of these lines remain flat and probably should not be counted as they fail to clear

We have clarified both the experimental design and how efficacy is defined. Phage was added when cultures reached early exponential phase (OD600 = 0.15), which explains the apparent delay in impact.

We have also expanded the explanation of the local *virulence index* (*vi*) and how this is used to define activity. Importantly, we now explicitly state that efficacy includes both medium and high local *vi* categories (*vi* ≥ 0.2), resulting in activity against 24/30 strains (80%). This is now more clearly justified in the Results and Table S3. Additional clarification was added in the Result section below:

The local vi score was categorised into low (local vi <0.2), medium (0.2 ≤ local vi ≥ 0.5), and high (local vi > 0.5). An effective killing is described as having medium and high local vi score (26). The initial version of the phage cocktail went through two iterations in our work (26), a phage cocktail consisting of phages 311F and 05F had 53 % (10/19) killing efficacy against ESBL-producing Klebsiella strains. In this study, we tested phages 311F and 05F against a larger set of clinically relevant ESBL-producing Klebsiella strains (Table 2) where it could target and kill 40% of tested isolates (12/30) (Figure 1b) (Table S3).

3. While the 6-phage cocktail did have activity on more strains than the 2-phage cocktail, there does seem to be evidence of antagonism that was not discussed (e.g., Fig 1d strains 13440 and KR311 showed that the 2- phage cocktail worked better than the 6-phage cocktail).

We agree this is an important point. We have now explicitly addressed both synergistic and antagonistic interactions in the Discussion. While the addition of four-phages increases overall breadth, We acknowledge that antagonism is observed for a subset of strains (e.g. KR311, 13440). We discuss potential mechanisms (e.g. receptor competition, interference between infection cycles) and emphasise that cocktail performance is strain dependent. An explanation was added in the discussion section:

*Comparison of 311F/05F and Leicester-UTI1 (Figure 1b, *p<0.05) revealed that the addition of four new phages (Centimanus, Storm, Anoxic, and SoFaint) significantly improved the coverage. Leicester-UTI1 demonstrated greater breadth of activity, targeting more strains than 311F/05F. A closer examination of individual bacterial strains suggests that the addition of phages is strain dependent. Synergistic effect was observed in most of the bacterial strains; however, antagonistic interaction was also identified, particularly in K. pneumoniae KR311, 13440, ARGID 36294, M201312995Q, and M201353511C. The addition of four-phages (Centimanus, Storm, Anoxic, and SoFaint) to 311F/05F appeared to reduce the killing efficacy against these strains, although the local vi score still indicated a medium killing activity (vi 0.20 - 0.50). The observed synergistic and antagonistic effects highlight that the growth and*

infection dynamics of one phage can influence those of others within the same bacterial host. Antagonistic effects or interference imply that one or more phages negatively impact the efficacy of others, potentially due to receptor competition or interference of lytic cycles (33). In contrast, synergistic interactions indicated an enhance key lytic phage characteristics, such as adsorption rate (infection efficiency), burst size (number of progeny produced), and lysis time (duration from infection to progeny release) (34).

4. The claim that no resistance to phage occurs in vivo is misleading because only 2 mice were tested using a Kp strain from a recurrent UTI patient which means this strain can hide out intracellularly, increasing the likelihood that they escape from the phages altogether. It would be nice to see EM images of bladder cells to assess where the Kleb cells are hanging out (inside cells?). It is not clear why many more samples were not tested for resistance or why spot testing was not performed to evaluate resistance. Why not test every sample for resistance? At least test Urine on day 5, which showed an uptick in bacterial growth from day 4.

   We agree that the original wording could be interpreted too strongly. As such we have revised the text to clarify the scope of the data. Resistance testing was performed on bladder isolates recovered at Day 7, where only two samples yielded colonies (Figure 2b). We acknowledge that additional temporal sampling would provide further insight and have now stated this clearly. We also note that this is being addressed in ongoing work using combination therapies and expanded sampling across timepoints.

   With respect to EM imaging, we agree this could provide additional insight into intracellular localisation. However, this falls outside the scope of the current study and is not aligned with the 3Rs framework guiding animal use. Instead, we emphasise that our conclusions are supported by bacterial burden, histology, and cytokine data, which together provide consistent evidence of infection and treatment response.

5. The figures are overly complex, making it difficult to read and distracting the reader from the main points. The figure layout in the biorxv is much better. Just do this as much as possible…

   - For Fig 1, I suggest removing 1a as this is not needed because this information is in the methods section. 1c can be removed or placed into supplemental data. That leaves 1b and d. Both can be made larger and the x-axis labels can be added to 1d (we need to see each hour of the growth curve).

   Thank you for the input, we tried to change the figures as suggested. Figure 1a was removed and 1c was added to Supplementary information (Figure S1).

   - Fig 2a can be moved to supplemental. Also, it is not clear what the efficacy figure is supposed to be or the chart under inflammation as they are not described in the legend.

   2b: The photo of the mice is not necessary and the rest of 2b can be moved to supplemental because this mouse model is not new. All that is needed in Fig 2 is 2c and d. It would be helpful to see where the phage was added in 2C (maybe with an arrow and a Ò?Ó symbol). 2e can also be moved to supplemental as it only shows that phage can infect the 3 recovered strains from 2 mice, and not well either.

Changed as suggested. Figure 2a. 2b, and 2e were all moved to Figure S3. An arrow symbol was added in 2a to indicate where the phage was added.

- Fig 3 should be split into 2 figures: 3a and b and then c would be figure 4. The bargraphs are just too small and the text on the x-axis is not ledgable. Also, the red color in the 3a right plot looks too brown. It needs to be the same colour as the legend.

Changed as suggested. Figure 3 was revised and Figure 3C was moved into Figure 4.

- I suggest combining Table1 content into Figure 3a+b as a key for the grading scale and remove Table 1.

Table 1 was moved as Figure 3a.

- Table 2 is ok

- Table 3 needs to have data for every K and O locus. Presumably, Kaptive was used to determine K and O loci? That is not mentioned anywhere in the paper! Any bacterial genomics and bioinformatics that was used needs to have text in the methods section. I also want to see the ESBL bla phenotypes or genotypes in Table 3 so the reader can determine that they are indeed ESBL and how diverse their AMR profiles are.

Table 3 was updated to provide a summary of the strains, while comprehensive K and O-locus analyses, performed using *Kleborate,* are available in Table S1. Comprehensive Antibiotic Resistance Database (CARD) was carried out and the results show 12/30 strains are ESBL-positive, predominantly carrying CTx-M-15 and less frequent SHV-12 and SHV-2A. Furthermore, 11/30 strains carry carbapenemase genes, including KPC-2 (n=7), NDM-1 (n=2), IMP-70 (n=1), VIM-1 (n=1). Notably, five-strains co-harbor both ESBL and carbapenemase determinants. Complete analyses are provided in Table S1-S2. In summary, these results highlight the diversity of the strain panel in terms of both surface antigens (K and O-loci) and antibiotic resistance profiles.

Further explanation based on this result was also added, as follows:

Sequence types (ST) of analysis confirms the diversity of the strains, with 22 distinct ST-types were identified (Table S1). Among these, ST147 – associated with hypervirulence and resistance plasmid (25) were observed in Leicester isolate (KR397) and Bristol isolate (P2.14) (Table S1). Capsule typing further revealed the diversity with distinct 25 K-loci and 7 O-loci (Table S1), indicating surface antigen variability. Genomic AMR predictions using CARD identified a total of 89 AMR determinants across the isolates (Figure S1b) (Table S2), with 40% (12/30) observed to carry ESBL (bla) genes, 36.7% (11/30) were carbapenems producer, and 5 strains harboured both (Table S1). This heterogeneity highlights the urgency for phage cocktails with broad host range. To address this, the Leicester-UTI1 phage cocktail (Table 1) was developed through a stepwise optimisation process.

Minor comments (line by line):

All minor comments have been addressed in the revised manuscript and are indicated in blue colored text in the manuscript.

1) lines 76,77: Add references to case reports using phage to treat Kp UTIs – revised.

Following reference was added and cited as Reference number 20: Kuipers S, Ruth MM, Mientjes M, Sévaux RGLd, Ingen Jv. A Dutch Case Report of Successful Treatment of Chronic Relapsing Urinary Tract Infection with Bacteriophages in a Renal Transplant Patient. Antimicrobial Agents and Chemotherapy. 2019;64(1):10.1128/aac.01281-19.

2) When citing reference 23, add more information about what that study provided, it is currently glossed over and insignificant. They used a 3-phage cocktail tested on a 70-strain panel of UTI clinical Kp strains. They also tested different MOIs and inoculation methods/routes which set the stage for your study (i.e., you didn't have to test MOI or inoculation route). Another distinction is that they inoculated with phage every day - revised, the following explanation was added in the text

Recent research has assessed the in vivo efficacy of phage therapy for Klebsiella pneumoniae UTIs, with the primary parameter being bacterial detection in urine samples (24). The study assessed 3-phage cocktail against a panel of 70 clinical K. pneumoniae strains in vitro. The cocktail was then tested in vivo through a different combination of administration route and range of multiplication of infections (MOIs). However, since the study only focuses on assessing bacterial load in urine samples, these results would not give information about the effects on intracellular bacterial communities (IBCs) or the pathology within the urinary tract.

3) line 92: italicize in vitro - revised

4) line 94,94-112: change obligatory to obligate and make sure that the order of figure references matches the order presented in the text. Currently Fig 1c is mentioned before b, before a. – the figures were moved as suggested and the text revised accordingly.

5) line 101: move Figure 1a to supplemental or remove entirely – revised, figure 1a was moved to the supplementary.

6) line 140,141: move Figure 2b to supplemental - revised

7) line 144: CFU/ml should be CFU/g as that was how you reported it in Fig 2d for bladder - revised

8) lines 148-150: Make the order of your experimental groups be the same as Fig2 and the Methods (Methods order is different) - revised

9) lines 163-170: To help the reader be confident that the implementation of this mouse model reflects human UTIs as much as possible, it would be useful to report any clinical symptoms that the mice had (e.g., fever, increase wbc, etc)? How do you know they have a UTI vs. asymptomatic bacteriuria?

Animals for the urinary tract model of infection, which are infected by the intraurethral or intravesical infection route, will not be expected to show signs from the infection as a whole. The aim is to use a low enough dose to produce colonisation without symptoms. The experiment was terminated 7 days post-infection, and mice were humanly killed. This is the scientific end point for this study. Infection may spread to the kidneys and cause systemic signs including septicemia; however, we didn't observe this effect in this study batch. This would not be expected for acute studies but could occur in more chronic studies. Additionally, we also showed through consistent bacterial counts in the bladder from 4-hours post infection up to Day-7 (Figure 2b), bladder histology showing the infected mice experienced bladder tissue inflammation (Figure 3c) as well

as increased pro-inflammatory cytokines starting from post-4 hours infection (Figure 4a-b), supporting the biomarker for UTIs.

10) line 172: change levels to abundance - revised

11) line 177: change in both infected groups to in both KLEB (or Klebsiella-infected) groupsÓ if that is what you mean here. Remember, you are infecting with 2 different things (bacteria and phage) - revised

12) lines 180-183: I really like this result and this needs to be discussed in the Discussion section. Singh et al used daily phage treatments in their study whereas you used only one. That is super important to know in the clinic. Also testing where the phage goes (bladder, kidney) and does not go (blood or spleen) is pharmacokinetics and this needs to be played up more in the Discussion and also in the abstract if room. Currently this important point is buried in the text -

    Additional explanation about comparative explanation with Singh et al study and the phage dynamic was added in the discussion section. The phage dynamics result was also added in the **Abstract** section.

    The additional explanations in the Discussions were as follows:

    Phage recovery and dynamics analysis further reveals that phage was able to actively replicate in the urinary lumen in which the bacteria presence. However, comparable phage titres were observed in the bladder between PHAGE and KLEB-PHAGE groups (Figure 2c), indicating limited intracellular phage replication. This is consistent with previous literatures depicting that host defence mechanism may limit phage multiplication intracellularly and thus preventing phage systemic circulation (50). Furthermore, no phage was detected in the blood or spleen also suggested that phage activity remains localised.

    Abstract was also updated with additional text:

    ..Phage recovery data indicate active replication in the urine, with no evidence of systemic circulation, as no phage was detected in the spleen or blood…

13) lines 193-201: This was mentioned in the Major concerns section of the review. More samples need to be tested (urine and bladder). Also include spot plaque assays.- answered in the 'Major Comment' section

14) Legend for Fig 3. State what **, ***, **** represent. Are they different P-values? - The P value that was used in the analysis is the same (p<0.05). Difference stars were used to indicate levels of significance value(*p<0.05, **p<0.01, ***p<0.001). A description was added in the figure 4 legend.

15) line 220: I think you want to say PBS CONTROL group served as a baseline as it does not make much sense to use the Klebsiella Control group (Fig 3a key) for baseline. Alternatively, you can clarify the key to Fig 3a with KLEB in red, KLEB-PHAGE in blue and PBS Control in green. Consistence of group labels across figures will make this read better - revised. Thank you for pointing this out. We changed the text to PBS Control (Baseline) and changed the color of the bar as well.

16) line 243: IFN-gamma, were to IFN-gamma were - revised

17) lines 239-262: Order of presentation. It would be easier to follow with Fig 3c (new Fig4) if you presented the bladder results first and then serum. Alternatively, just change the order in Fig 3c to match the order presented in the text - revised

18) line 269,270: Establishing a mouse model does not address the gap as multiple mouse models exist (e.g., ref 26 and 23 and others not cited). The gap being addressed is testing the efficacy of the improved 6-phage cocktail in vivo. – revised as below

To address this gap, our study aimed to provide comprehensive evaluation of both the efficacy and safety of Klebsiella-targeting phage cocktail, Leicester-UTI1, in a robust in vivo model.

19) line 294,295: target as many as possible clinical strains to target as many clinical strains as possible - revised

20) line 302: add in vivo after bacterial growth - revised

21) line 303: please clarify the synergy statement since 13440 and KR311 both seemed to have antagonism in the 6-phage cocktail compared to the 2-phage cocktail - Following explanation was added in the discussion section

*Comparison of 311F/05F and Leicester-UTI1 (Figure 1b, *p<0.05) revealed that the addition of four new phages (Centimanus, Storm, Anoxic, and SoFaint) significantly improved the coverage. Leicester-UTI1 demonstrated greater breadth of activity, targeting more strains than 311F/05F. A closer examination of individual bacterial strains suggests that the addition of phages is strain dependent. Synergistic effect was observed in most of the bacterial strains; however, antagonistic interaction was also identified, particularly in K. pneumoniae KR311, 13440, ARGID 36294, M201312995Q, and M201353511C. The addition of four-phages (Centimanus, Storm, Anoxic, and SoFaint) to 311F/05F appeared to reduce the killing efficacy against these strains, although the local vi score still indicated a medium killing activity (vi 0.20 - 0.50). The observed synergistic and antagonistic effects highlight that the growth and infection dynamics of one phage can influence those of others within the same bacterial host. Antagonistic effects or interference imply that one or more phages negatively impact the efficacy of others, potentially due to receptor competition or interference of lytic cycles (33). In contrast, synergistic interactions indicated an enhance key lytic phage characteristics, such as adsorption rate (infection efficiency), burst size (number of progeny produced), and lysis time (duration from infection to progeny release) (34).*

22) lines 307-312: ÒIndeed, genomic analysis (Table 3)ÉÓ This seems to be ghost text from a previous version perhaps as no genomic analysis was mentioned in the Methods or Results sections and no depolymerase data is presented in Table 3. Either remove this text or add the data to the manuscript - we refer to the previously published paper, and an explanation was provided in the discussion as below

Previous genomic analysis conducted by Jameson et. al (27) provided additional insights, confirming that phage Centimanus in Leicester-UT1 encodes nine depolymerase proteins.

23) line 325: in murine to in a murine - revised

24) line 327: mimicking acute human bladder infection. As far as I can tell, no clinical data (e.g. body temp/fever, white blood cell count, etc) was presented to suggest nothing more than asymptomatic bacteriuria

Animals for the urinary tract model of infection, which are infected by the intraurethral or intravesical infection route, will not be expected to show signs from the infection as a whole. The aim is to use a low enough dose to produce colonisation without symptoms. The experiment was terminated 7 days post-infection, and mice were humanly killed. This is the scientific end point for this study. Infection may spread to the kidneys and cause systemic signs including septicaemia, however we didn't observe this effect in this study batch. This would not be expected for acute studies but could occur in more chronic studies. UTIs marker was also indicated by the presence of high (CFU > 10^4/ ml) bacterial burden in the bladder, histology data (Figure 3b) and pro-inflammatory cytokines (Figure 4).

25) line 328: I would argue that this strain Top52 might not be the best for strain for acute UTI as it seems to be more appropriate in a model of recurrent UTI since it came from a patient with recurrent UTI and you presented supporting data that it can hide out in the bladder, presumably escaping phage attack. Again, an EM of these cells would show Kleb inside cells.

Kp Top52 has been used in the previous studies (Ref 41 and 42 in the text), we based our choice from this research as a proof of concept. Indeed, we show data that Top52 can hide in the bladder even on Day-7. This would be a great suggestion to investigate longer study duration for recurrent UTI or chronic UTI which was already mentioned in our study limitation and suggestion.

26) line 366,367: Perhaps discuss determining which of the 6 phages cause IL-10 induction? Do you see that with the 2-phage cocktail - We did not test 2-phages cocktail in the mice; therefore, we don't have the cytokine samples for this.

27) It would be intellectually useful to discuss 2 vs 3 vs 6-phage cocktail in a paragraph also discussing the difference between Singh et al and this study. Multiple doses are not required for the 6-phage cocktail here but maybe for 2 or 3-phage cocktails? These are important factors to consider when developing a therapeutic phage cocktail.

Additional explanation was added in the discussion section (paragraph 8), depicting the comparative result with Singh *et al* study and highlighting a broader scope of our study.

…. This finding is consistent with previous work by Singh et al (24), which demonstrated the efficacy of single administration of phage therapy application through the urethra in a murine UTI model. This result also suggests that the Leicester-UTI1 cocktail is more effective in an in vivo system than in vitro. Notably, our observation extends beyond the scope of the existing result (24), suggesting that the phage may effectively target IBCs within the bladder. We hypothesise that the observed efficient clearance of bacteria is a consequence of phage activity in the localised urinary systems and the synergistic interactions with the murine immune response, thus the phages have driven an efficient infection resolution. …

28) under limitations: Please add that the adaptive immune response was not evaluated (e.g., IgA, IgG). Only 2 mice and 3 colonies of recovered Kleb were evaluated for phage resistance. Only 30 strains were tested *in vitro* and that an expanded panel needs to be screened to determine the effectiveness of the 6-phage cocktail in the clinic. It would be useful to have host range data of each phage independently as supplemental data to determine if all 6 phages are required.

Checking for adaptive immune response has been added as a limitation as well as future suggestion (Discussion, line 391 - 393). The host range of single phage was already available at Table S1. Currently, our clinical strains panel has been representative as it was sourced from diverse clinical collections across the UK (England and Wales) and represented UK ST types.

29) line 415: Luria Bertani is the incorrect name according to Bertani, 2004 (PMC321500). It is supposed to be called Lysogeny Broth. If the authors feel strongly to keep Luria Bertani, I suggest hyphenating it as Luria-Bertani – revised. We changed Luria Bertani to 'Luria-Bertani'

30) line 429: please italicize a priori - revised

31) line: 441: mmMgSO4 should be mm MgSO4 - revised

32) Phage host range PKA section. Was each phage added in equal amounts to the cocktail in a ratio of 1:1:1:1:1:1?

Yes, information added into the method

33) lines 492-495: please make sure the group number order matches the text and Figs - revised.

34) line 526: Were yellow colonies picked? On CLED, yellow colonies should be Klebsiella - a description was added in the method

Klebsiella pneumoniae Top52 colonies (yellow coloured) were isolated from bladder samples of Group 2 KLEB-PHAGE.

35) line 529: Cocktail 2 should be LEICESTER-UTI1 no? - revised

36) line 530: MOI 1 to MOI of 1 - revised

37) line 532: DASH should be Dashing Growth Curves according to https://dashing-growth-curves.ethz.ch/ - revised

38) Phage susceptibility testing post murine infection. Please state at what timepoint and how many mice were tested (2 according to the text). Also, did you really test urine samples for phage resistance? If so, which timepoint(s)?

We changed the detailed description to:

Klebsiella pneumoniae Top52 colonies (yellow coloured) were isolated from bladder samples of Group 2 KLEB-PHAGE on Day 7.

39) references 10 and 11 need to have a space after 10. And 11. - revised

40) references: please italicize all bacteria genus and species names. Ref 23 for sure needs Klebsiella pneumoniae to be in italics. - revised

41) Are any of the panel strains hypermucoid?

Yes, Kp 30104 is hypermucoid strain. Information was added in Table 2 and supported by genomic annotation that shows Kp30104 *rmpA2* genes that can produce mucus. Information available in theTable S1.

42) Do you observe any phage resistance in vitro? Kp is notorious for rapidly obtaining phage resistance

We did not test any resistance *in vitro,* so we are unable to determine if it occurred *in vitro.*

43) I recommend changing the title by removing LEICESTER-UT1 phage to a 6-phage

We think that giving the cocktail a name will make the text easier to follow.

Reviewer #3 (Comments for the Author):

This manuscript presents a rigorous evaluation of the Leicester-UTI1 phage cocktail as a therapeutic strategy against uropathogenic Klebsiella pneumoniae, including ESBL-producing clinical isolates. The authors demonstrate strong in vitro efficacy across a clinically relevant panel and validate these findings in a mouse model that closely mimics acute urinary tract infection. Phage treatment significantly reduced bacterial burden in urine, bladder, and kidney within hours of administration, without evidence of resistance emergence. The additional analysis of inflammatory markers further strengthens the translational relevance of the work. The data are compelling,, and the conclusions are well supported.

Only minor comment is that the text within several figures appears somewhat small and difficult to read.

Overall, this is an excellent and clinically relevant study that makes a meaningful contribution to the field of phage therapy.

We thank the reviewer for their positive and encouraging assessment of the work.

In response to the comment regarding figure readability, we have revised all figures to improve clarity, including resizing panels, simplifying layouts, and moving supporting data to the Supplementary Information.

Overall, we believe these revisions have significantly improved the clarity, balance, and presentation of the manuscript, while addressing the reviewer’s concerns in full.

Re: Spectrum03952-25R1 (**Development and *in-vivo* efficacy of LEICESTER-UTI1 phage cocktail that targets ESBL-associated *Klebsiella* from urinary tract infections**)

Dear Dr. Martha R.J. Clokie:

Your manuscript has been accepted, and I am forwarding it to the ASM production staff for publication. Your paper will first be checked to make sure all elements meet the technical requirements. ASM staff will contact you if anything needs to be revised before copyediting and production can begin. Otherwise, you will be notified when your proofs are ready to be viewed.

Data Availability: ASM policy requires that data be available to the public upon online posting of the article, so please verify all links to sequence records, if present, and make sure that each number retrieves the full record of the data. If a new accession number is not linked or a link is broken, provide production staff with the correct URL for the record. If the accession numbers for new data are not publicly accessible before the expected online posting of the article, publication may be delayed; please contact ASM production staff immediately with the expected release date.

Publication Fees: For information on publication fees and which article types have charges, please visit our website. We have partnered with Copyright Clearance Center (CCC) to collect author charges. If fees apply to your paper, you will receive a message from no-reply@copyright.com with further instructions. For questions related to paying charges through RightsLink, please contact CCC at ASM_Support@copyright.com or toll free at +1-877-622-5543. CCC makes every attempt to respond to all emails within 24 hours.

ASM Membership: Corresponding authors may join or renew ASM membership to obtain discounts on publication fees. Need to upgrade your membership level? Please contact Customer Service at Service@asmusa.org.

Embargo Policy: A press release may be issued as soon as the manuscript is posted on the Spectrum Latest Articles webpage. The corresponding author will receive an email with the subject line "ASM Journals Author Services Notification" when the article is available online.

The ASM Journals program strives for constant improvement in our submission and publication process. Please tell us how we can improve your experience by taking this quick Author Survey.

Thank you for submitting your paper to Spectrum.

Sincerely,
David Pride
Editor
Microbiology Spectrum

Reviewer #3 (Comments for the Author):

The authors have satisfactorily addressed the previously raised comments and concerns.
