# Supplementary material for: Development and in-vivo efficacy of Leicester-UTI1 phage cocktail that targets ESBL-associated Klebsiella from urinary tract infections
Source: Microbiol Spectr. 2026 Jun 24;14(8):e03952-25. doi: 10.1128/spectrum.03952-25 (PMC13436347; doi:10.1128/spectrum.03952-25)
Supplement: Supplementary file 2 [file spectrum.03952-25-s0001.docx]

Supplementary Data

a.

b.

Figure S1 Genomic analysis of *Klebsiella* isolates used in this study. a) Phylogenetic analysis associated with Sequence Types (ST), Capsular (K) and O-Antigen loci b) A heatmap of AMR predictions based on the Comprehensive Antibiotic Resistance Database (CARD) identified 89 AMR determinants across the isolates, complete analysis in Table S2.

Figure S2 Phylogenetic tree of six-*Klebsiella* phages in Leicester-UTI1 cocktail was constructed with VipTree and visualised using iTOL(v6)

Supplementary figure 1. Killing curve of *K.pneumoniae* Top52 against Purified or non-purified Phage cocktail at MOI 1. The purified cocktail still has the same killing effect as the non-purified. The local virulence index (*vi)* was 0.37 and 0.35, for purified and non-purified phage cocktail, respectively.

Figure S3. Killing curve of *K.pneumoniae* Top52 against Purified or non-purified Phage cocktail at MOI 1. The purified cocktail still has the same killing effect as the non-purified. The local virulence index (*vi)* was 0.37 and 0.35, for purified and non-purified phage cocktail, respectively.

Figure S4. Phage cocktail assessment *in vivo* a) Experimental design to assess efficacy and safety of 126 Leicester-UTI1 phage cocktail b) Establishment of an acute UTI murine model using K. pneumoniae Top52 via transurethral inoculation c) Growth curve from recovered K. pneumoniae Top52 colonies (Original Top52, Rec 191, Rec 197 and 134 Rec 200) then challenged with Leicester-UTI1; no significant difference in the growth rate (Table S4) *and local vi* score 0.30, 0.32, 0.34 and 0.34, respectively.

**Baseline**

Score: 0

***Klebsiella* untreated**

Score: 2

Score: 3	Score: 5	Score: 4

Score: 5	Score: 4	Score: 5

***Kleb-Phage***

Score: 1	Score: 4

Score: 5	Score: 3	Score: 1

Score: 1	Score: 2	Score: 4

Figure S5. Histology pictures from the histology of bladder samples. From top – bottom: baseline, *Klebsiella* untreated, and *Kleb-phage*.

| **Phage Name**  Data S1. List of phages used in this study. | **TEM** | **Genus** | **Genome Size** | **Reference** |
| --- | --- | --- | --- | --- |
| vB_KpnM_311F |  | *Jiaodavirus* | 186 kb | 34 |
| vB_KpnM_05F |  | *Jiaodavirus* | 175 kb | 34 |
| vB_KpM_Centimanus (Phage 71) |  | *Maaswegvirus* | 299 kb | - |
| vB_KppS_Storm (Phage 34) |  | *Sugarlandvirus* | 110 kb | 36 |
| vB_KppS_Anoxic (Phage 52) |  | *Sugarlandvirus* | 109 kb | 36 |
| vB_KpM_SoFaint (Phage 70) |  | *Slopekvirus* | 176 kb | 36 |

**Result:**

Data S2. Endotoxin Quantification from Purified Phage Lysate

Information S1. Script used to analyse local *vi* (Rstudio, 2025)

#install the package -

install.packages("DescTools")

#load the lib

library("DescTools")

###read in the data from norm sheet

library(readxl)

testdata <- read_excel("Template R - Copy of K016.xlsx",

sheet = "Norm")

View(testdata)

attach (testdata)

#plot the c-nve samples to file

pdf("c-nve_plot.pdf")

plot(G10, col="red")

points (G11, col="green")

points (G12, col="purple")

dev.off()

###plot to screen

plot(G10, col="red")

points (G11, col="green")

points (G12, col="purple")

#plot the blank samples to file

pdf("blank_plot.pdf")

plot(H10, col="red")

points (H11, col="green")

points (H12, col="purple")

dev.off()

##plot blank to screen

plot(H10, col="red")

points (H11, col="green")

points (H12, col="purple")

#plot strain A

plot(A7, col="red" , log ="y", ylim=c(0.01,5) )

points (A8, col="green")

points (A9, col="purple")

plot(B7, col="red" , log ="y", ylim=c(0.01,5) )

points (B8, col="green")

points (B9, col="purple")

#strainc

plot(C7, col="red" , log ="y", ylim=c(0.01,5) )

points (C8, col="green")

points (C9, col="purple")

#strainD

plot(D7, col="red" , log ="y", ylim=c(0.01,5) )

points (D8, col="green")

points (D9, col="purple")

#strainE

plot(E7, col="red" , log ="y", ylim=c(0.01,5) )

points (E8, col="green")

points (E9, col="purple")

#strainF

plot(F7, col="red" , log ="y", ylim=c(0.01,5) )

points (F8, col="green")

points (F9, col="purple")

#strain G

plot(G7, col="red" , log ="y", ylim=c(0.01,5) )

points (G8, col="green")

points (G9, col="purple")

#strainH

plot(H7, col="red" , log ="y", ylim=c(0.01,5) )

points (H8, col="green")

points (H9, col="purple")

#need to define the period of flattening off from the above graphs

#AUC curve by strains

#define the last time point

last = "50"

###this might change on the strain !!!!!

##strainA

####strain A cocktail 1 AUC

auc.A1cock1.1 <-AUC(Time[1:last], A1[1:last])

auc.A1cock1.2 <-AUC(Time[1:last], A2[1:last])

auc.A1cock1.3 <-AUC(Time[1:last], A3[1:last])

##strain a cocktail 2 AUC

auc.A1cock2.1 <-AUC(Time[1:last], A4[1:last])

auc.A1cock2.2 <-AUC(Time[1:last], A5[1:last])

auc.A1cock2.3 <-AUC(Time[1:last], A6[1:last])

##control strain A

auc.A1control.1 <-AUC(Time[1:last], A7[1:last])

auc.A1control.2 <-AUC(Time[1:last], A8[1:last])

auc.A1control.3 <-AUC(Time[1:last], A9[1:last])

####strain B cocktail 1 AUC

auc.B1cock1.1 <-AUC(Time[1:last], B1[1:last])

auc.B1cock1.2 <-AUC(Time[1:last], B2[1:last])

auc.B1cock1.3 <-AUC(Time[1:last], B3[1:last])

##strain B cocktail 2 AUC

auc.B1cock2.1 <-AUC(Time[1:last], B4[1:last])

auc.B1cock2.2 <-AUC(Time[1:last], B5[1:last])

auc.B1cock2.3 <-AUC(Time[1:last], B6[1:last])

##control strain B

auc.B1control.1 <-AUC(Time[1:last], B7[1:last])

auc.B1control.2 <-AUC(Time[1:last], B8[1:last])

auc.B1control.3 <-AUC(Time[1:last], B9[1:last])

####strain C cocktail 1 AUC

auc.C1cock1.1 <-AUC(Time[1:last], C1[1:last])

auc.C1cock1.2 <-AUC(Time[1:last], C2[1:last])

auc.C1cock1.3 <-AUC(Time[1:last], C3[1:last])

##strain C cocktail 2 AUC

auc.C1cock2.1 <-AUC(Time[1:last], C4[1:last])

auc.C1cock2.2 <-AUC(Time[1:last], C5[1:last])

auc.C1cock2.3 <-AUC(Time[1:last], C6[1:last])

##control strain C

auc.C1control.1 <-AUC(Time[1:last], C7[1:last])

auc.C1control.2 <-AUC(Time[1:last], C8[1:last])

auc.C1control.3 <-AUC(Time[1:last], C9[1:last])

####strain D cocktail 1 AUC

auc.D1cock1.1 <-AUC(Time[1:last], D1[1:last])

auc.D1cock1.2 <-AUC(Time[1:last], D2[1:last])

auc.D1cock1.3 <-AUC(Time[1:last], D3[1:last])

##strain D cocktail 2 AUC

auc.D1cock2.1 <-AUC(Time[1:last], D4[1:last])

auc.D1cock2.2 <-AUC(Time[1:last], D5[1:last])

auc.D1cock2.3 <-AUC(Time[1:last], D6[1:last])

##control strain D

auc.D1control.1 <-AUC(Time[1:last], D7[1:last])

auc.D1control.2 <-AUC(Time[1:last], D8[1:last])

auc.D1control.3 <-AUC(Time[1:last], D9[1:last])

####strain E cocktail 1 AUC

auc.E1cock1.1 <-AUC(Time[1:last], E1[1:last])

auc.E1cock1.2 <-AUC(Time[1:last], E2[1:last])

auc.E1cock1.3 <-AUC(Time[1:last], E3[1:last])

##strain E cocktail 2 AUC

auc.E1cock2.1 <-AUC(Time[1:last], E4[1:last])

auc.E1cock2.2 <-AUC(Time[1:last], E5[1:last])

auc.E1cock2.3 <-AUC(Time[1:last], E6[1:last])

##control strain E

auc.E1control.1 <-AUC(Time[1:last], E7[1:last])

auc.E1control.2 <-AUC(Time[1:last], E8[1:last])

auc.E1control.3 <-AUC(Time[1:last], E9[1:last])

####strain F cocktail 1 AUC

auc.F1cock1.1 <-AUC(Time[1:last], F1[1:last])

auc.F1cock1.2 <-AUC(Time[1:last], F2[1:last])

auc.F1cock1.3 <-AUC(Time[1:last], F3[1:last])

##strain F cocktail 2 AUC

auc.F1cock2.1 <-AUC(Time[1:last], F4[1:last])

auc.F1cock2.2 <-AUC(Time[1:last], F5[1:last])

auc.F1cock2.3 <-AUC(Time[1:last], F6[1:last])

##control strain F

auc.F1control.1 <-AUC(Time[1:last], F7[1:last])

auc.F1control.2 <-AUC(Time[1:last], F8[1:last])

auc.F1control.3 <-AUC(Time[1:last], F9[1:last])

####strain G cocktail 1 AUC

auc.G1cock1.1 <-AUC(Time[1:last], G1[1:last])

auc.G1cock1.2 <-AUC(Time[1:last], G2[1:last])

auc.G1cock1.3 <-AUC(Time[1:last], G3[1:last])

##strain G cocktail 2 AUC

auc.G1cock2.1 <-AUC(Time[1:last], G4[1:last])

auc.G1cock2.2 <-AUC(Time[1:last], G5[1:last])

auc.G1cock2.3 <-AUC(Time[1:last], G6[1:last])

##control strain G

auc.G1control.1 <-AUC(Time[1:last], G7[1:last])

auc.G1control.2 <-AUC(Time[1:last], G8[1:last])

auc.G1control.3 <-AUC(Time[1:last], G9[1:last])

####strain H cocktail 1 AUC

auc.H1cock1.1 <-AUC(Time[1:last], H1[1:last])

auc.H1cock1.2 <-AUC(Time[1:last], H2[1:last])

auc.H1cock1.3 <-AUC(Time[1:last], H3[1:last])

##strain H cocktail 2 AUC

auc.H1cock2.1 <-AUC(Time[1:last], H4[1:last])

auc.H1cock2.2 <-AUC(Time[1:last], H5[1:last])

auc.H1cock2.3 <-AUC(Time[1:last], H6[1:last])

##control strain H

auc.H1control.1 <-AUC(Time[1:last], H7[1:last])

auc.H1control.2 <-AUC(Time[1:last], H8[1:last])

auc.H1control.3 <-AUC(Time[1:last], H9[1:last])

#######################################################

#local vi values per strain

#strainA

vi.A1cock1.1 <-1-(auc.A1cock1.1/auc.A1control.1)

vi.A1cock1.2 <-1-(auc.A1cock1.2/auc.A1control.2)

vi.A1cock1.3 <-1-(auc.A1cock1.3/auc.A1control.3)

vi.A1cock2.1 <-1-(auc.A1cock2.1/auc.A1control.1)

vi.A1cock2.2 <-1-(auc.A1cock2.2/auc.A1control.2)

vi.A1cock2.3 <-1-(auc.A1cock2.3/auc.A1control.3)

#strainB

vi.B1cock1.1 <-1-(auc.B1cock1.1/auc.B1control.1)

vi.B1cock1.2 <-1-(auc.B1cock1.2/auc.B1control.2)

vi.B1cock1.3 <-1-(auc.B1cock1.3/auc.B1control.3)

vi.B1cock2.1 <-1-(auc.B1cock2.1/auc.B1control.1)

vi.B1cock2.2 <-1-(auc.B1cock2.2/auc.B1control.2)

vi.B1cock2.3 <-1-(auc.B1cock2.3/auc.B1control.3)

#strainC

vi.C1cock1.1 <-1-(auc.C1cock1.1/auc.C1control.1)

vi.C1cock1.2 <-1-(auc.C1cock1.2/auc.C1control.2)

vi.C1cock1.3 <-1-(auc.C1cock1.3/auc.C1control.3)

vi.C1cock2.1 <-1-(auc.C1cock2.1/auc.C1control.1)

vi.C1cock2.2 <-1-(auc.C1cock2.2/auc.C1control.2)

vi.C1cock2.3 <-1-(auc.C1cock2.3/auc.C1control.3)

#strainD

vi.D1cock1.1 <-1-(auc.D1cock1.1/auc.D1control.1)

vi.D1cock1.2 <-1-(auc.D1cock1.2/auc.D1control.2)

vi.D1cock1.3 <-1-(auc.D1cock1.3/auc.D1control.3)

vi.D1cock2.1 <-1-(auc.D1cock2.1/auc.D1control.1)

vi.D1cock2.2 <-1-(auc.D1cock2.2/auc.D1control.2)

vi.D1cock2.3 <-1-(auc.D1cock2.3/auc.D1control.3)

#strainE

vi.E1cock1.1 <-1-(auc.E1cock1.1/auc.E1control.1)

vi.E1cock1.2 <-1-(auc.E1cock1.2/auc.E1control.2)

vi.E1cock1.3 <-1-(auc.E1cock1.3/auc.E1control.3)

vi.E1cock2.1 <-1-(auc.E1cock2.1/auc.E1control.1)

vi.E1cock2.2 <-1-(auc.E1cock2.2/auc.E1control.2)

vi.E1cock2.3 <-1-(auc.E1cock2.3/auc.E1control.3)

#strainF

vi.F1cock1.1 <-1-(auc.F1cock1.1/auc.F1control.1)

vi.F1cock1.2 <-1-(auc.F1cock1.2/auc.F1control.2)

vi.F1cock1.3 <-1-(auc.F1cock1.3/auc.F1control.3)

vi.F1cock2.1 <-1-(auc.F1cock2.1/auc.F1control.1)

vi.F1cock2.2 <-1-(auc.F1cock2.2/auc.F1control.2)

vi.F1cock2.3 <-1-(auc.F1cock2.3/auc.F1control.3)

#strainG

vi.G1cock1.1 <-1-(auc.G1cock1.1/auc.G1control.1)

vi.G1cock1.2 <-1-(auc.G1cock1.2/auc.G1control.2)

vi.G1cock1.3 <-1-(auc.G1cock1.3/auc.G1control.3)

vi.G1cock2.1 <-1-(auc.G1cock2.1/auc.G1control.1)

vi.G1cock2.2 <-1-(auc.G1cock2.2/auc.G1control.2)

vi.G1cock2.3 <-1-(auc.G1cock2.3/auc.G1control.3)

#strainH

vi.H1cock1.1 <-1-(auc.H1cock1.1/auc.H1control.1)

vi.H1cock1.2 <-1-(auc.H1cock1.2/auc.H1control.2)

vi.H1cock1.3 <-1-(auc.H1cock1.3/auc.H1control.3)

vi.H1cock2.1 <-1-(auc.H1cock2.1/auc.H1control.1)

vi.H1cock2.2 <-1-(auc.H1cock2.2/auc.H1control.2)

vi.H1cock2.3 <-1-(auc.H1cock2.3/auc.H1control.3)

####mean values

strainA.cock1.mean <- mean(c(vi.A1cock1.1,vi.A1cock1.2,vi.A1cock1.3))

strainA.cock2.mean <- mean(c(vi.A1cock2.1,vi.A1cock2.2,vi.A1cock2.3))

strainA.cock1.mean <- mean(c(vi.A1cock1.1,vi.A1cock1.2,vi.A1cock1.3))

strainA.cock2.mean <- mean(c(vi.A1cock2.1,vi.A1cock2.2,vi.A1cock2.3))

strainB.cock1.mean <- mean(c(vi.B1cock1.1,vi.B1cock1.2,vi.B1cock1.3))

strainB.cock2.mean <- mean(c(vi.B1cock2.1,vi.B1cock2.2,vi.B1cock2.3))

strainC.cock1.mean <- mean(c(vi.C1cock1.1,vi.C1cock1.2,vi.C1cock1.3))

strainC.cock2.mean <- mean(c(vi.C1cock2.1,vi.C1cock2.2,vi.C1cock2.3))

strainD.cock1.mean <- mean(c(vi.D1cock1.1,vi.D1cock1.2,vi.D1cock1.3))

strainD.cock2.mean <- mean(c(vi.D1cock2.1,vi.D1cock2.2,vi.D1cock2.3))

strainE.cock1.mean <- mean(c(vi.E1cock1.1,vi.E1cock1.2,vi.E1cock1.3))

strainE.cock2.mean <- mean(c(vi.E1cock2.1,vi.E1cock2.2,vi.E1cock2.3))

strainE.cock1.mean <- mean(c(vi.E1cock1.1,vi.E1cock1.2,vi.E1cock1.3))

strainE.cock2.mean <- mean(c(vi.E1cock2.1,vi.E1cock2.2,vi.E1cock2.3))

strainF.cock1.mean <- mean(c(vi.F1cock1.1,vi.F1cock1.2,vi.F1cock1.3))

strainF.cock2.mean <- mean(c(vi.F1cock2.1,vi.F1cock2.2,vi.F1cock2.3))

strainG.cock1.mean <- mean(c(vi.G1cock1.1,vi.G1cock1.2,vi.G1cock1.3))

strainG.cock2.mean <- mean(c(vi.G1cock2.1,vi.G1cock2.2,vi.G1cock2.3))

strainH.cock1.mean <- mean(c(vi.H1cock1.1,vi.H1cock1.2,vi.H1cock1.3))

strainH.cock2.mean <- mean(c(vi.H1cock2.1,vi.H1cock2.2,vi.H1cock2.3))

##sd values

strainA.cock1.sd <- sd(c(vi.A1cock1.1,vi.A1cock1.2,vi.A1cock1.3))

strainA.cock2.sd <- sd(c(vi.A1cock2.1,vi.A1cock2.2,vi.A1cock2.3))

strainA.cock1.sd <- sd(c(vi.A1cock1.1,vi.A1cock1.2,vi.A1cock1.3))

strainA.cock2.sd <- sd(c(vi.A1cock2.1,vi.A1cock2.2,vi.A1cock2.3))

strainB.cock1.sd <- sd(c(vi.B1cock1.1,vi.B1cock1.2,vi.B1cock1.3))

strainB.cock2.sd <- sd(c(vi.B1cock2.1,vi.B1cock2.2,vi.B1cock2.3))

strainC.cock1.sd <- sd(c(vi.C1cock1.1,vi.C1cock1.2,vi.C1cock1.3))

strainC.cock2.sd <- sd(c(vi.C1cock2.1,vi.C1cock2.2,vi.C1cock2.3))

strainD.cock1.sd <- sd(c(vi.D1cock1.1,vi.D1cock1.2,vi.D1cock1.3))

strainD.cock2.sd <- sd(c(vi.D1cock2.1,vi.D1cock2.2,vi.D1cock2.3))

strainE.cock1.sd <- sd(c(vi.E1cock1.1,vi.E1cock1.2,vi.E1cock1.3))

strainE.cock2.sd <- sd(c(vi.E1cock2.1,vi.E1cock2.2,vi.E1cock2.3))

strainE.cock1.sd <- sd(c(vi.E1cock1.1,vi.E1cock1.2,vi.E1cock1.3))

strainE.cock2.sd <- sd(c(vi.E1cock2.1,vi.E1cock2.2,vi.E1cock2.3))

strainF.cock1.sd <- sd(c(vi.F1cock1.1,vi.F1cock1.2,vi.F1cock1.3))

strainF.cock2.sd <- sd(c(vi.F1cock2.1,vi.F1cock2.2,vi.F1cock2.3))

strainG.cock1.sd <- sd(c(vi.G1cock1.1,vi.G1cock1.2,vi.G1cock1.3))

strainG.cock2.sd <- sd(c(vi.G1cock2.1,vi.G1cock2.2,vi.G1cock2.3))

strainH.cock1.sd <- sd(c(vi.H1cock1.1,vi.H1cock1.2,vi.H1cock1.3))

strainH.cock2.sd <- sd(c(vi.H1cock2.1,vi.H1cock2.2,vi.H1cock2.3))

vector.export <-c(strainA.cock1.mean, strainA.cock1.sd, strainA.cock2.mean, strainA.cock2.sd, strainB.cock1.mean, strainB.cock1.sd, strainB.cock2.mean, strainB.cock2.sd, strainC.cock1.mean, strainC.cock1.sd, strainC.cock2.mean, strainC.cock2.sd, strainD.cock1.mean, strainD.cock1.sd, strainD.cock2.mean, strainD.cock2.sd, strainE.cock1.mean, strainE.cock1.sd, strainE.cock2.mean, strainE.cock2.sd, strainF.cock1.mean, strainF.cock1.sd, strainF.cock2.mean, strainF.cock2.sd, strainG.cock1.mean, strainG.cock1.sd, strainG.cock2.mean, strainG.cock2.sd, strainH.cock1.mean, strainH.cock1.sd, strainH.cock2.mean, strainH.cock2.sd)

write.table(vector.export,file="Means_sd_values.txt",quote=FALSE, sep = "\t ",
